# KIF15 promotes human glioblastoma progression under the synergistic transactivation of REST and P300

**DOI:** 10.7150/ijbs.98668

**Published:** 2024-09-23

**Authors:** Wendan Yu, Shilong Han, Sheng Hu, Liyuan Ru, Chunyu Hua, Guoqing Xue, Guohui Zhang, Kuan Lv, Hanxiao Ge, Meiyi Wang, Lina Zheng, Jie Zhou, Shuai Hou, Yun Teng, Wuguo Deng, Wei Guo

**Affiliations:** 1 Institute of Cancer Stem Cell & The Second Affiliated Hospital, Dalian Medical University, Dalian, China.; 2 Sun Yat-sen University Cancer Center; State Key Laboratory of Oncology in South China; Collaborative Innovation Center of Cancer Medicine, Guangzhou 510060, China.; 3 Guangdong Provincial Hospital of Integrated Traditional Chinese and Western Medicine, Guangdong, China.

**Keywords:** Glioblastoma (GBM), KIF15, REST, P300, Acetylation

## Abstract

Glioblastoma (GBM) is highly invasive and lethal. The failure to cure GBM highlights the necessity of developing more effective targeted therapeutic strategies. KIF15 is a motor protein to be involved in cell mitosis promotion, cell structure assembly and cell signal transduction. The precise biological function and the potential upstream regulatory mechanisms of KIF15 in GBM remain elusive. Here, we demonstrated that KIF15 was abnormally up-regulated in GBM and predicted poor prognosis of GBM patients. KIF15 promotes GBM cell proliferation, metastasis and cell cycle progression. REST could bind to KIF15 promoter and transactivate KIF15. Furthermore, REST interacts with P300 and depends on its histone acetyltransferase (HAT) activity to co-regulate KIF15 expression. Both REST and P300 were highly expressed in GBM and predicted poor prognosis of GBM patients alone or in combination with KIF15. The tumorigenic function of KIF15 in GBM was regulated by REST *in vitro* and *in vivo* and the combinational treatment of cell cycle inhibitor Palbociclib with P300 HAT inhibitor inhibited GBM xenografts survival more significantly. Our findings indicate that KIF15 promotes GBM progression under the synergistic transactivation of REST and P300. P300/REST/KIF15 signaling axis is expected to be served as a cascade of candidate therapeutic targets in anti-GBM.

## Introduction

As the most commonly and highly malignant brain tumor according to WHO classification criteria 1, glioblastoma accounts for 12-15% of all intracranial tumors and even constitutes 50%-60% of Astrocytoma 2. Once diagnosed as GBM, an estimated median survival time of patients is approximately only 1 year. The main strategies for GBM treatments are surgery, radiotherapy and chemotherapy. Highly proliferative and invasive property 3, tumor heterogeneity and high angiogenesis ability of GBM 4, 5 coupled with the drug's lower blood-brain barrier transmissibility make GBM the most aggressive tumor with a poor prognosis. Thus, identifying and validating the key regulators involved in GBM progression and exploring and developing more efficient targeted therapeutic strategy accordingly is urgently needed.

KIF15 is one member of Kinesin-12 family proteins and is essential for the normal physiological processes. KIF15 has been reported to interact with C-terminal domain of TPX2 to control microtubule-based movement 6. It has also been referred to maintain neuronal development 7. During prometaphase, KIF15 is needed to separate spindle and to maintain spindle bipolarity at metaphase by interacting with KBP 8. But in the pathology condition, especially in carcinogenesis and development, KIF15 dysregulation has been reported to act as tumorigenic factor by promoting cancer cell proliferation and migration, including pancreatic cancer 9, prostate cancer 10, colorectal cancer 11 and nasopharyngeal carcinoma 12. Furthermore, KIF15 knockdown induces G1/S phase cell cycle arrest and its expression is positively associated with poor prognosis in patients with lung adenocarcinoma 13. However, its precise function and the underlying molecular mechanisms in GBM evolution remain unclear. Considering the essential tumor-promoting role of KIF15 in different cancer types mentioned above, the exploration and revealing of the functions and the potential regulatory mechanisms of KIF15 in glioblastoma progression seem to be a necessity.

During neurodevelopment, some transcription factors have been reported to restrict the expressions of neuronal genes in non-neuronal cells to ensure neuron development. Repressor element-1 silencing transcription factor (REST), also known as neuron-restrictive silencer factor (NRSF), is one of such factors. REST regulates various target genes involved in neuronal differentiation 14, axonal growth 15, and vesicular transport and release 16. Altered REST expression is not only found in mental disorder, neurobehavioral disorders 17-19 and ischemic stroke 20, but also found to be highly correlated with brain tumors. Especially in nervous system cancer, REST often plays tumor-promoting roles. In neuroblastoma cells, REST is found to be reduced during cell differentiation 21. In medulloblastoma, REST drives cancer progression via epigenetic modification and AKT activation 22. Additionally, the elevated REST promotes migration and vascular growth of medulloblastoma cells 23, 24. Mechanistically, the transcriptional regulation of REST requires the assembly of multimeric complex. For example, in non-neuronal cells, REST is shown to repress neuronal gene expression by recruiting the SIN3/HDAC complex 25 or recruiting G9a and interacting with Mediator via MED19/MED26 and MED12 subunits 26. Although REST has been identified to drive tumor growth in GBM via its transcription activity 27, its precise downstream regulatory targets and more detailed transcriptional regulatory mechanisms remain elusive and deserve to be better focused on and explored.

Chromatin modifications play a crucial role in the regulation of gene expression. As a histone acetyltransferase, P300 can acetylate histones, a common but key epigenetic event, to reduce chromatin condensation and subsequently participate in the transcription process 28. Besides, P300 can also regulate transcription by acting as a coactivator of transcription to cooperate with other transcription factors dependent on or independent of its acetyltransferase activity 29, 30. Here, in this study, based on ChIP-seq database, we found the binding peak of P300 at the specific promoter region of KIF15 gene in GBM cells, which was also proved to be bound by REST. We therefore further explored and clarified the possible synergistic regulation of REST and P300 on KIF15 transcription in GBM cells and the dependence of such synergy on the histone acetyltransferase activity of P300. The clinical significance of P300/REST/KIF15 signaling axis in GBM, their sequential promotion on cell cycle progression and cell survival, and their partial or total inactivation in anti-GBM alone or in combination with cell cycle inhibitor, were also explored and revealed.

## Methods and materials

### Cell culture and reagents

All cells were purchased from the American Type Culture Collection. The glioblastoma cell lines, U87MG, U118MG, SHG44, A172 and LN229 were maintained with Dulbecco's Modified Eagles Medium (Gibco) and T98G was cultured in Eagle's Minimum Essential Medium (Gibco). The complete medium was supplied with 10% fetal bovine serum and 1% penicillin and streptomycin. All cells were cultured in 37°C incubator with 5% CO_2_. U0126 (#9903) and LY294002 (#9901) were purchased from Cell Signaling Technology. C646 (#T2452) was purchased from TargetMol (Shanghai, China) and Palbociclib (#HY-50767) was purchased from MedChemExpress (Shanghai, China).

### siRNAs and Plasmids

The KIF15 siRNAs were purchased from GenePharm (Shanghai, China) with the following sequences: siRNA2 (5'-AAAGGAAACUCUUUCCAGCTT-3'; 5' -GCUGGAAAGAGUUUCCUUUTT-3'), and siRNA3 (5'-UUCAGAUCCUGCUAAAUCCTT-3'; 5'-GGAUUUAGCAGG AUCUGAATT-3'). pGIPZ-shNC, pGIPZ-shKIF15, pGIPZ-shREST plasmids were purchased from Thermo Scientific CCSB-Broad lentiviral expression Library. pEZ-LV206-REST plasmid and NEG-LV206 plasmid were purchased from GeneCopoeia (Guangzhou, China). pCMV-KIF15 overexpression plasmid (#P56000) was purchased from MiaoLing Biology (Wuhan, China). P300 wild type, P300-mutant, flag-P300 full length and truncate mutation plasmids were kindly provided by Prof. Tiebang Kang.

### Primers and antibodies

Primers for KIF15 (forward: 5'-AAAACTGAGTTACGCAGCGTG-3', reverse: 5'-AGTTGCGAATACAGATTCCTGAG-3'), primers for REST (forward: 5' -TTTCTCCAAGGGCCCCATTC-3', reverse: 5'-GTCTTCTGAGAACTTGAGTAAGGAC-3') and primers for P300 (forward: 5'-TATGATCCGTGGCAGTGTGC-3', reverse: 5'-TAGGTACAGGCG AGGGTGAA-3') were synthesized from Sangon Biotech in Shanghai. Antibodies against KIF15 (55407-1-AP), REST (22242-1-AP), β-actin (20536-1-AP), N-cadherin (22018-1-AP), Flag (66008-2-lg), Rabbit IgG (30000-0-AP), GAPDH (60004-1-lg) were purchased from proteintech (Wuhan, China). p-c-Raf (#9427), Erk1/2 (#4695), p-Erk1/2 (#4370), p-AKT(S_473_) (#4060), AKT (#4691), Vimentnin (#5741), Snail (#3789), Slug (#9585), Cyclin A2 (#91500), CyclinD1 (#2978), CyclinD3 (#2936), CDK2 (#2546), CDK6 (#3136), p-cdc2 (#9111), p-Rb(S_807/811_) (#8516), p-Rb(S_780_) (#8180), p21 (#2947), p18INK4C (#2896), Histone H3 (#4499), Ki67 (#9449) were purchased from Cell Signaling Technology (MA, USA). P300 (sc-48343), and mouse IgG (sc-515946) were purchased from Santa Cruz (Shanghai, China).

### Database analysis

KIF15 expression in 12 different cancers was analyzed by Oncomine database (https://www.oncomine.org/resource/login.html). The expression of KIF15, REST and P300 in GBM compared to normal tissues was identified by Human Protein Atlas (https://www.proteinatlas.org/). Expression profiles of mRNA data of glioblastoma were downloaded from Gene Expression Omnibus (GEO) database (http://www.ncbi.nl m.nih.gov/geo/), and dataset GSE4290 was used to analyze the expression of KIF15 in normal and different cancer stages of glioblastoma. The correlation of KIF15 between cell cycle marker and REST expression in glioblastoma were analyzed using TCGA database. The expression of REST in normal tissues and GBM tissues, the correlation of REST with P300 expression in GBM, the overall survival in GBM and LGG patients with different REST, KIF15 and P300 expression were analyzed by Gene Expression Profiling Interactive Analysis (GEPIA) database (http://gepia.cancer-pku.cn/). The recognition motif of REST in mammalian cells was downloaded in JASPAR databases (https://jaspar.elixir.no/). P300 and REST's binding site at the loci of KIF15 promoter were predicted by WashU epigenome browser in ChIP-seq databases (https://epigenomegateway.wustl.edu/browser/). The interaction between P300 and REST was predicted by STRING databases (https://string-db.org/).

### Cell viability assay

Cells were plated in 96-well plates and cultured overnight. The drugs, siRNAs or plasmids were used to treat cells with different doses and desired time. 10% MTT was added to the cell with continuous culture for four hours. The absorbance value at 490 nm was measured and the cell viability was calculated.

### Colony formation assay

A total of 500-1000 cells were plated into 6-well plate overnight. Cells were transfected with KIF15 specific siRNAs or control siRNAs. After 24 h, cells were treated with DMSO or palbociclib and then cultivated for 14 days. The cells were fixed with the mixture (methanol: glacial: acetic=1:1:8) for 15 min, washed twice with PBS and then stained with 0.1% crystal violet/10% ethanol for 20 minutes. Colony were photographed and calculated.

### Gap-closure assay

The cells were transfected with siRNAs and maintained to 90% -100% confluence in 6-well plates. Then the cell monolayers were scratched using a 200 ul sterile pipette tip. The wounds were incubated further and the images were taken at time 0 hour, 24 hours, 48 hours, and 72 hours respectively. The migration rates were quantified.

### Transwell assay

The cell invasion ability was performed using 24-well chemotaxis chambers (corning, USA). The cells were plated in six-well plates and transfected with siRNAs, plasmids or treated with drugs. Then the cells were collected and resuspended with serum-free medium and 5×10^4^ cells were added to the upper chamber coated with a mixture of serum free medium and Matrigel (BD Biosciences, USA). The lower chamber was filled with 500 ul medium with 20% fetal serum. The cells were incubated for 48 h or 72 h in the upper chamber. Then the cells located at the filter were washed with PBS twice and fixed with 4% paraformaldehyde for 10min and then stained with 0.1% crystal violet for 15 min. Images of the cell were visualized by inverted microscope and the cells were counted by Image J software.

### Confocal immunofluorescence assay

The cells were plated on the cover glass in the six-well plate. After culture overnight, the cells were fixed with 4% paraformaldehyde for 10 minutes and permeabilized with 0.2% Triton X-100 and then blocked with 10% bovine serum albumin (BSA) for 30 min. After blocking, cells were incubated with KIF15 (1:50 dilution), or P300 (1:50 dilution) and REST (1:200 dilution) antibody overnight at 4°C. After three times washing with PBS, cells were incubated with FITC-or Rhodamine-conjugated secondary antibody for 1 hour in the dark room. Subsequently, the nuclei were stained with DAPI for five minutes. After 3 times washing with each for 10 min, the images were visualized by Leica DM1400B confocal laser scanning microscope.

### Co-immunoprecipitation assay

Flag-P300-WT, flag-P300-F1 to F5 plasmids were transfected into U87MG cells for 96 h or 48 h, and then cells were lysed in immunoprecipitation buffer (50 mM Tris-HCl pH 8.0, 150 mM NaCl, 0.05 mM EDTA, 1% NP40 and 10% glycerol) and the lysate was pre-cleared with protein A/G agarose beads (Santa Cruz, Shanghai) for 2 h at 4°C. The supernatant were immunoprecipitated overnight with anti-flag or REST antibody and subsequently immunoprecipitated with protein A/G agarose for 4 h at 4°C. The pellets were washed three times with IP buffer, and then resuspended in loading buffer followed by SDS-PAGE analysis.

### Cell cycle analysis

After transfection with siRNA and plasmids or treatment with drugs, cells were collected and fixed with ice-cold 75% ethanol overnight at -20°C. The fixed cells were stained with 0.5 ml of propidium iodide (PI) buffer containing 100 ug/ml RNase A, 0.2% Triton X-100 and 50 ug/ml PI at 37°C for 30 min in the dark. The stained cells were analyzed by BD Accuri C6 plus flow cytometry.

### DNA-pulldown assay

The probe was synthesized by PCR using biotin-labeled primers from Sangong Company. After being transfected with plasmids or treated with inhibitors, the nucleus proteins of the cells were mixed with biotinylated KIF15 promoter probe (4 ug), streptavidin agarose beads (50 ul) in 500 ul PBSI buffer containing 1 mM PMSF, 0.5 M NaF, 2.5 mM β-glycerophosphate, 1 mM Na_3_VO_4_, 0.1 mM DTT, 20 uM Leupeptin and rotated for 4 h at room temperature. After centrifugation and washing with PBSI for three times, the beads were resuspended with 1×loading buffer and boiled at 100°C for 10 min. The supernatant was analyzed by Western blot.

### Chromatin Immunoprecipitation (ChIP) assay

Briefly, U87MG or T98G cells were transfected with shRNAs, overexpression plasmids or treated with drugs for the desired time, and then fixed with 1% paraformaldehyde, sonicated on ice to shear the DNA into 200-500 bp fragments. A small portion of cell lysate was used as DNA input control, and the remaining lysate was subjected to immunoprecipitations with anti-REST or anti-P300 antibody. The non-specific rabbit or mouse IgG was used as negative control. The immunoprecipitated DNA was subjected to PCR to amplify a fragment of KIF15 promoter with the length of 116 bp. The primers used for PCR was as following: 5'-GTGAAAAGAGGGACCCGACA-3' and 5'-CGTAGAGACTAAGGTGAGC-3'.

### Animal study

All the animal maintenance and operational procedures were carried out according to the recommendations in the Guide for the Care and Use of Laboratory Animals approved by Animal Care and Ethics Committee of Dalian Medical University. Four-six week old high immune deficiency NYG mice were purchased from Liaoning Changsheng Biotechnology Co., Ltd. For the rescue experiment, the human glioblastoma cell U87MG with stable knockdown of KIF15 and/or overexpression of REST was established by lentivirus infection. The mice were randomly divided into four groups (n=6 for each group): LacZ+shNC, LacZ+shKIF15, REST OE+shNC, REST OE+shKIF15, and the corresponding cells (1×10^7^ suspended in 150 ul PBS) were injected subcutaneously into right flank of each mice. Two weeks after injection, the tumor diameters were measured once every two days and tumor volume was calculated as V= (Width^2^×lengh)/2. Mice were sacrificed and tumors were taken from mice for weighting and photographing. For the drug treatment experiment, 1×10^7^ U87MG cells resuspended in 150 ul PBS were injected into the flank of each NYG mice. Mice were randomly divided into four groups: vehicle, palbociclib treatment, C646 treatment and combination treatment (n=6 for each group), and received different treatments at 6 days after cell injection. Palbociclib (75 mg/kg) was given orally once a day, C646 (8 mg/kg) was injected intraperitoneally once a day, and the combination group was administrated with drugs every other day for 10 days respectively. The tumor diameters and mice weight were measured every two days. One week after the end of drug treatment, the mice were sacrificed and the blood was collected for blood urea nitrogen (BUN) analysis. The tumors were removed, weighted at the final time point, and partial of them was analyzed by Western blot, while the rest of them were fixed with 10% formalin for IHC analysis.

### Immunohistochemistry staining

The xenograft tumors were dissected and fixed with 10% formalin overnight, embedded in paraffin, and incised to 4µm thickness. Immunohistochemistry staining was performed using PV9000 kit (ZSGB-BIO, China) according to instructions. Briefly, the sections were blocked with blocking reagents, incubated with antibodies respectively against REST (1:800 dilution), KIF15 (1:100 dilution), p-RB (1:800 dilution), CyclinD1 (1:50 dilution), Slug (1:50 dilution), p-AKT (Ser473) (1:50 dilution), or Ki67 (1:400 dilution) at 4°C overnight. After being washed with PBS and incubated with anti-mouse/rabbit biotin-labeled secondary antibody, the slides were stained with DAB kit (ZSGB-BIO, China), and counterstained with hematoxylin. Finally, the slides were dehydrated using gradient alcohol, sealed with neutral balsam (Solarbio, China) and photographed with microscope.

### Statistical analysis

Data were represented as mean± SD from at least three independent experiments. Statistical analysis between two samples was performed using Student's t-test. Statistical comparisons of more than two groups were performed using one-way analysis of variance (ANOVA). All statistical analyses were performed using Graph Pad Prism 8.0 software (SanDiego, USA). P<0.05 was considered statistically significant.

## Results

### KIF15 is highly expressed in glioblastoma and promotes cancer cell proliferation and metastasis

**Figure 1:**
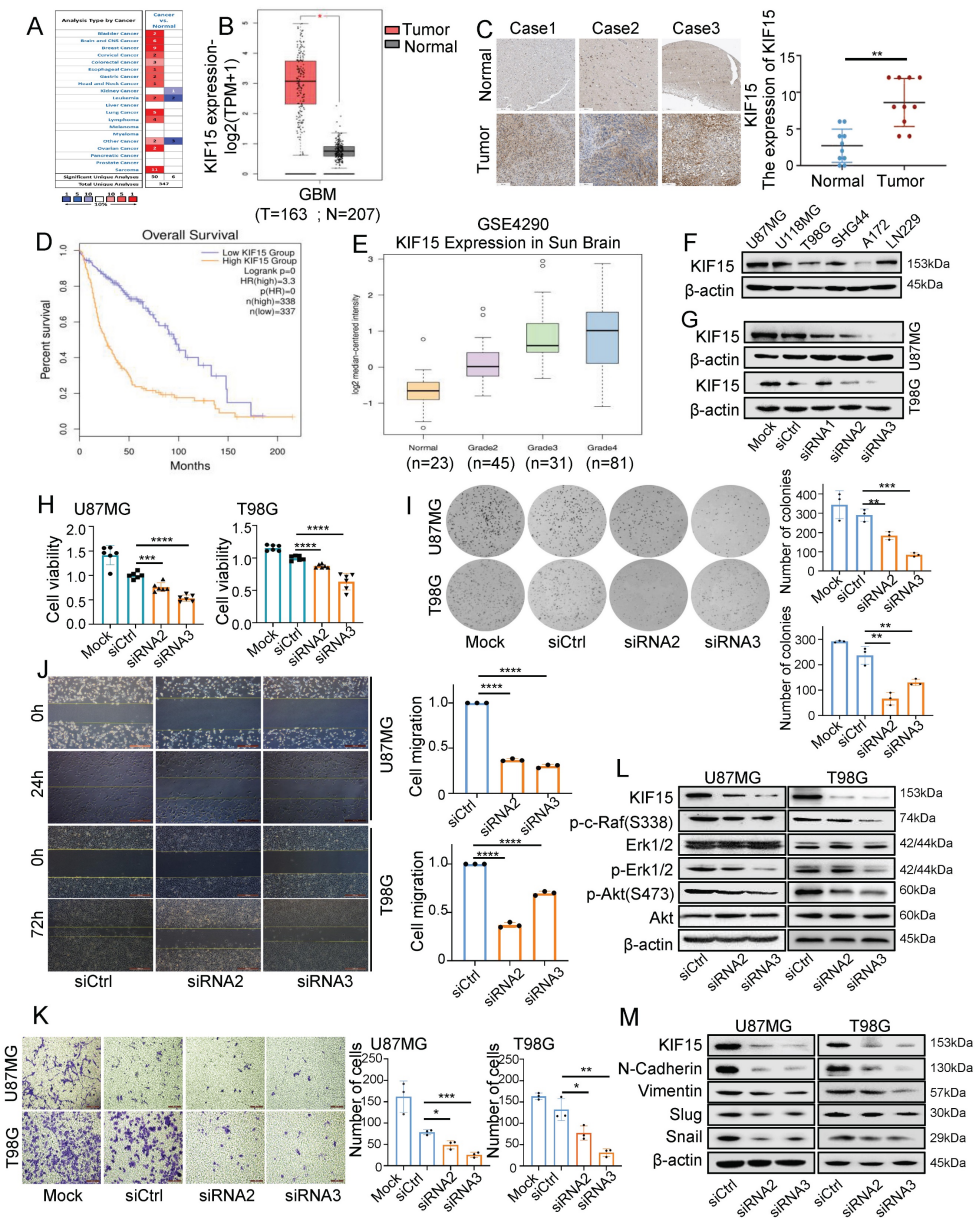
** KIF15 is highly expressed in glioblastoma and promotes cancer cell proliferation and metastasis.** (**A**) KIF15 is highly expressed in 12 kinds of different cancers based on Oncomine database, including Brain and CNS Cancer. (**B**) KIF15 expression is dramatically upregulated in glioblastoma compared to normal brain tissue in TCGA and GTEx databases. (**C**) KIF15 expression in three cases of brain tumors and the corresponding adjacent normal brain tissues and quantified presentation of its expression according to the IHC score. (**D**) The overall survival of GBM patients with KIF15 high and low expression was analyzed according to GEPIA databases. (**E**) The expression of KIF15 increases with the grade of glioma, and it's the highest in WHO grade IV glioblastoma in GEO datasets. (**F**) KIF15 expression in different glioblastoma cell lines was detected by western blot. (**G**) KIF15 expression was knocked down by its specific siRNAs and detected by western blot in U87MG and T98G cells. (**H**) Cell viability was detected by MTT assay after cells were cultured without transfection (Mock) or transfected with non-specific control (siCtrl), or KIF15 specific siRNAs respectively. (**I-K**) Colony formation assay (**I**), gap-closure assay (**J**) and transwell assay (**K**) of U87MG and T98G cells transfected with KIF15 specific siRNAs for 48 hours and the corresponding statistical analysis. (**L**) Western blot assay for expression of p-c-Raf, Erk1/2, p-Erk1/2, p-AKT (s473), AKT and β-actin in U87MG and T98G cells after KIF15 were knocked down. (**M**) Western blot assay for the expression of N-cadherin, Vimentin, Slug and Snail in U87MG and T98G cells after KIF15 was knocked down. The data represent the mean±SD of three independent experiments, and the level of significance was indicated by *P<0.05, **P<0.01, ***P<0.001, ****P<0.0001. Mock: blank control. siCtrl: control siRNA.

In order to evaluate the functions of KIF15 in glioblastoma progression, we first analyzed its expression based on clinical data from Oncomine and Human protein Atlas database. Compared to normal tissues, we found that KIF15 was highly expressed in 12 different kinds of cancers (Fig. 1A), especially in glioblastoma (Fig. 1B, C). We further acquired the overall survival information of glioblastoma patients with different KIF15 expression in TCGA&GTEx database, and the result showed that high KIF15 expression predicted poor outcomes in glioblastoma patients (Fig. 1D). By analyzing the mRNA expression data (GSE4290) from GEO database, the expression of KIF15 was gradually up-regulated with the increase of pathologic grade in glioblastoma (Fig. 1E).

We also analyzed the expression of KIF15 in different glioblastoma cell lines (Fig.1F). U87MG and T98G cells were chosen to knockdown KIF15 for their abundant expression of KIF15 and higher transfection efficiency (Fig. 1G). Immunofluorescence assay indicated that KIF15 localizes in both cytoplasma and nucleus (Supplementary Fig. 1A). After knocking down KIF15, cell viability, colony formation ability and cell migration and invasion capacity were all significantly inhibited (Fig. 1H-K). Furthermore, we detected AKT, Erk and EMT pathway-related proteins upon KIF15 knockdown. The results showed that the expressions of p-c-Raf, p-Erk1/2, p-Akt(S473), N-Cadherin, Vimentin, Slug, and Snail were all accordingly decreased after KIF15 was knocked down (Fig. 1L, M). These results collectively suggested that KIF15 might play a crucial tumor-promoting role in GBM by maintaining cancer cell proliferation and metastasis.

To further confirm the key contribution of Erk or Akt signaling pathway activation to the proliferative function of KIF15 in GBM cells, we treated U87MG and T98G cells with Erk or Akt pathway inhibitor alone or combined with KIF15 siRNA. MTT assay showed that cell viability was significantly decreased upon treatment with U0126/LY294002 or KIF15 siRNA. But compared to inhibitor and control siRNA co-treatment group, cell viability was not obviously decreased upon co-treatment with inhibitor and KIF15 siRNA (Supplementary Fig. 1B, C), whereas, inhibitor treatment obviously alleviated KIF15 overexpression-mediated cell viability promotion, which is indicated by the statistical analysis that no significant difference was observed between control and KIF15 overexpression group after inhibitor treatment (Supplementary Fig. 1E, F). These results demonstrate that, Erk or AKT signaling pathway plays a significant role in KIF15-mediated growth promotion in GBM cells.

### KIF15 promotes cell cycle progression and its knockdown sensitizes glioblastoma cells to palbociclib treatment

**Figure 2:**
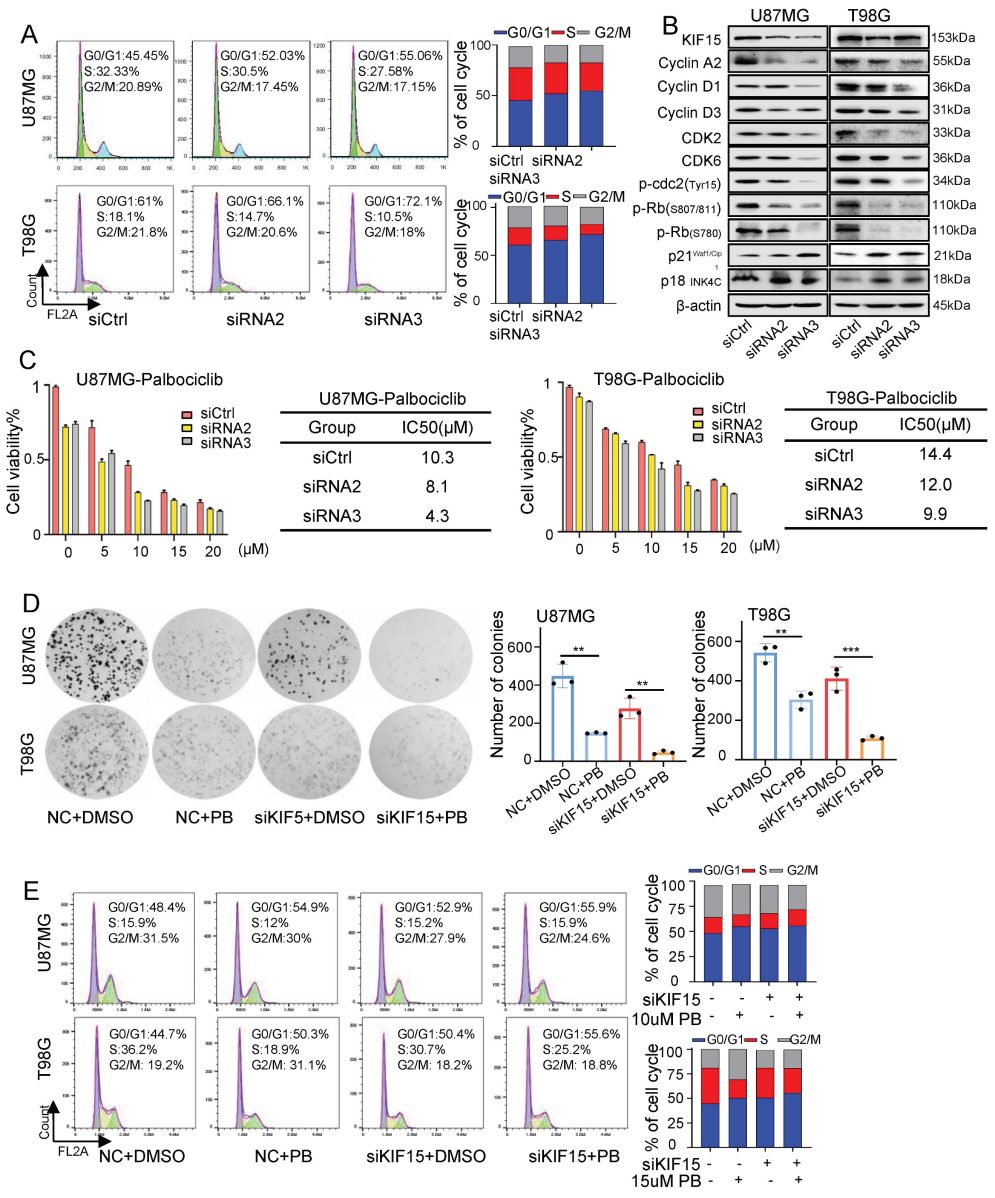
**Knockdown of KIF15 induces G0/G1 phase arrest and sensitizes glioblastoma cells to Palbociclib treatment.** (**A**) Representative cell cycle plots of U87MG and T98G cells at 48 h after KIF15 knockdown. (**B**) The expressions of KIF15 and cell cycle-associated proteins were analyzed by western blot after KIF15 was knocked down for 48 h. (**C**) U87MG and T98G cells were transfected with KIF15 specific siRNAs or control siRNAs for 24 h, and then were treated by different concentration of Palbocilib for another 24 h. Cell viability was determined by MTT assay and IC50s were calculated. (**D-E**) Colony formation (**D**) and cell cycle assay (**E**) were performed respectively in U87MG and T98G cells with KIF15 knockdown and treatment of 10µM or 15µM Palbocilib sequentially. The corresponding quantified analysis was also performed. The data represents the mean±SD of three independent experiments, and the level of significance was indicated by *P<0.05, **P<0.01, ***P<0.001. NC: Negative control. PB: Palbociclib.

Considering the essential role of KIF15 in cell mitosis 8, we deduced its tumor-promoting function in GBM might be realized by its regulation on cell cycle. The percentages of cells at each cell cycle phase were analyzed in U87MG and T98G cells by flow cytometry upon KIF15 knockdown. After transfection with KIF15 specific siRNAs, compared to control group, GBM cells accumulated more at G0/G1 phase, which was accompanied by a concomitant reduction in the proportion of cells in S and G2/M phase (Fig. 2A). Meanwhile, western blot assay in these two cells demonstrated that KIF15 knockdown significantly reduced the protein levels of Cyclin A2, CyclinD1, CyclinD3, CDK2, CDK6, and p-Rb, which are crucial for cells entering into S phase from G0/G1 phase, whereas cyclin-dependent kinase inhibitor P21 Waf1/CIP and p18INK4C were enhanced upon KIF15 silencing (Fig. 2B). These results demonstrated that KIF15 silencing induced G0/G1 cell cycle arrest in glioblastoma cells. By analyzing the expression correlation between KIF15 and cell cycle-related genes in 528 glioblastoma patients, KIF15 expression was found to be highly positively correlated with CCNA2, CDC25A and CDK2, moderately positively correlated with ANAPC1, CCND1 and CDK4, and weakly positively correlated with P300, CDK6 and RB1 (Supplementary Fig. 2A,B). These results supported our hypothesis that KIF15 promotes glioblastoma cell growth by accelerating cell cycle progression.

Furthermore, we combined CKD4/6 inhibitor Palbociclib with KIF15 siRNAs to evaluate whether KIF15 knockdown could increase GBM cell sensitivity to Palbociclib. MTT assay were used to determine the IC50 value of Palbociclib in U87MG and T98G cells. Compared with control group, the IC50 value was decreased from 10.3 µM to 8.1 µM and 4.3 µM in U87MG cells, and from 14.4 µM to 12 µM and 9.9 µM in T98G cells respectively upon KIF15 silencing (Fig. 2C). Colony formation assay was also performed to evaluate the influence of KIF15 knockdown on cell growth after Palbociclib treatment. KIF15 silencing markedly improved cell growth inhibition caused by Palbociclib (Fig. 2D). We further knocked down KIF15 expression using its specific siRNAs in U87MG and T98G cells and performed cell cycle analysis in such cells with or without Palbociclib treatment. The results showed that KIF15 knockdown combined with Palbociclib treatment induced more G0/G1 phase arrest when compared to single treatment (Fig. 2E). Furthermore, we performed another MTT assay to assess the role of cell cycle in KIF15-mediated GBM cell function by using CDK4/6 inhibitor, Palbociclib treatment. As shown in Supplementary Fig. 1D and 1G, Palbociclib co-treatment with KIF15 siRNA alleviated KIF15 knockdown-mediated cell viability inhibition (Fig. S1D). Meanwhile, Palbociclib treatment reversed the growth promotion by KIF15 overexpression in GBM cells (Fig. S1G). All these results indicated that cell cycle pathway plays crucial roles in KIF15-mediated growth promotion and provided the potential that KIF15 silencing synergizes with cell cycle inhibitor to produce more significant anti-cancer effect in GBM treatment.

### REST binds to KIF15 promoter and transactivates KIF15 to promote glioblastoma progression *in vitro* and* in vivo*

**Figure 3:**
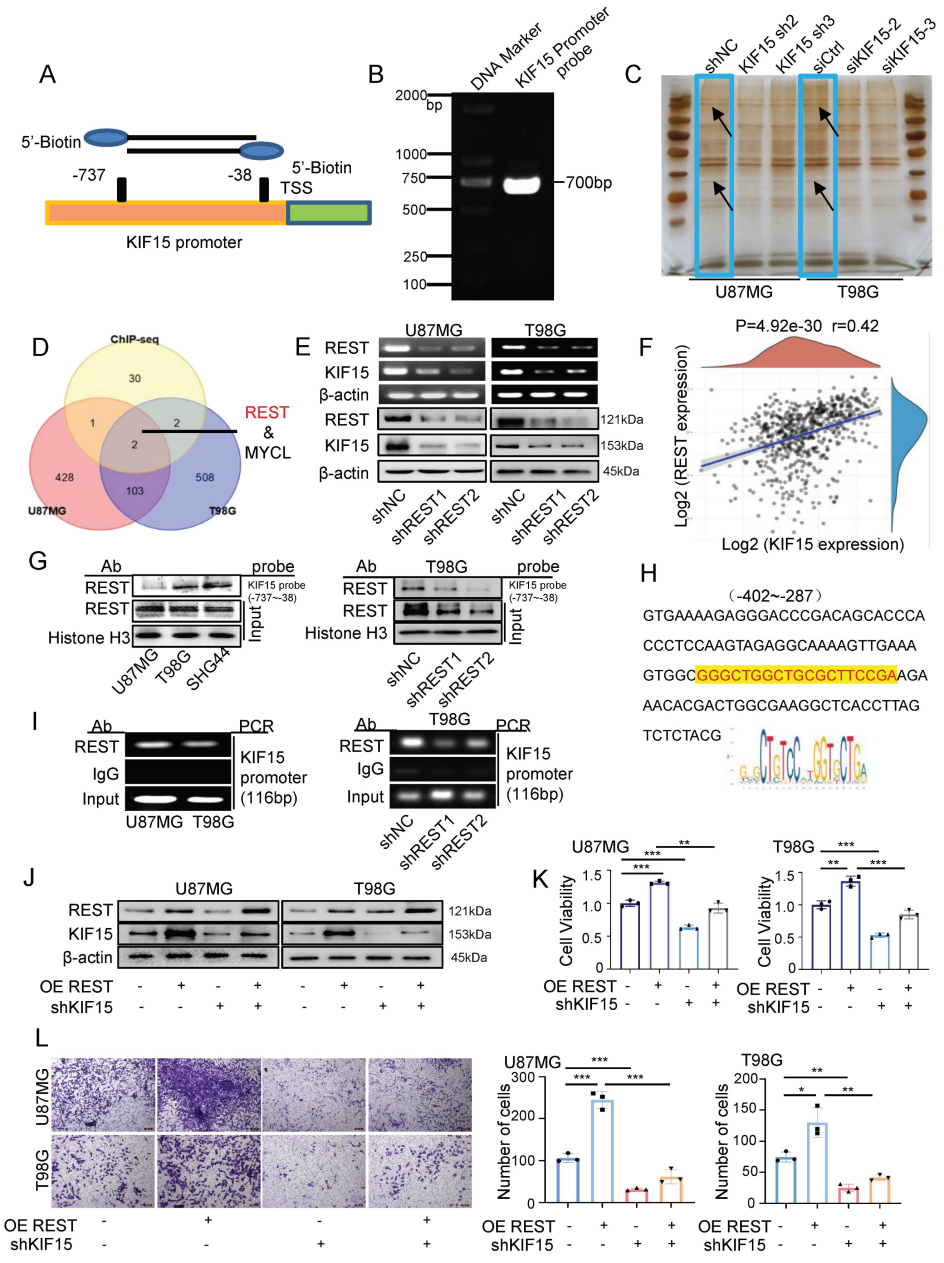
** REST binds to KIF15 promoter and transactivates KIF15 to promote glioblastoma progression *in vitro*.** (**A**) Schematic diagram of biotinylated KIF15 promoter probe was shown. (**B**) Biotin-labeled KIF15 promoter probe was synthesized by PCR. (**C**) Silver staining analysis of KIF15 promoter-binding proteins separated by SDS-PAGE. (**D**) The overlaps of the pulled-down proteins by KIF15 promoter probe in U87MG and T98G cells and the binding proteins at KIF15 promoter region in these two cells from ChIP-seq data. (**E**) Expressions of REST and KIF15 in U87MG and T98G cells after silencing REST with its specific shRNAs were measured by RT-PCR and Western blot. (**F**) The positive correlation between REST and KIF15 expression was analyzed in TCGA database based on their mRNA level. (**G**) REST was pulled down by KIF15 promoter probe in different GBM cells or in T98G cells with REST knockdown by DNA-pulldown assay. (**H**) The binding sequence recognized by REST in KIF15 promoter. (**I**) ChIP assay was performed using REST specific antibody or IgG to detect KIF15 promoter sequence from -402 to -287 (product length=116 bp) in different GBM cells or in T98G cells with REST knockdown. (**J**) The expressions of REST and KIF15 were determined by Western blot in U87MG and T98G cells after REST was overexpressed and/or KIF15 was knocked down. (**K**) MTT assay was performed in U87MG and T98G cells with REST overexpression and/or KIF15 knockdown. (**L**) Transwell assay was performed in U87MG and T98G cells with REST overexpression and/or KIF15 knockdown, and the invasive cells were photographed and calculated. The data represent the mean±SD of three independent experiments, and the level of significance was indicated by *P<0.05, **P<0.01, ***P<0.001.

Given the essential tumor-promoting role of KIF15 in glioblastoma cell survival, it is meaningful to delineate the underlying mechanisms controlling KIF15 expression. We performed DNA-pulldown assay to screen and identify its potential transcription factors. The biotin-labeled KIF15 promoter probe was designed and synthesized firstly for pulldown experiment (Fig. 3A, B). The nuclear proteins binding to the probe were separated by SDS-PAGE and silver-stained before mass spectrometry (Fig. 3C). The identified proteins were subjected to intersection analysis and preliminary screening with the results of ChIP-seq from the Cistrome DB database, and REST protein was chosen and focused on as the candidate regulator of KIF15 (Fig. 3D). We then verified the positive regulation of REST on KIF15 expression at mRNA and protein levels in U87MG and T98G cells (Fig. 3E). Consistently, a positive correlation between REST and KIF15 expression in glioblastoma tissues from TCGA database was observed (Fig. 3F). In order to further confirm the possible transcriptional regulation of REST on KIF15, considering its own property as a transcription factor, we next analyzed the binding of REST on KIF15 promoter. DNA pulldown assay confirmed that REST could bind to KIF15 promoter probe in different glioblastoma cells and such binding was alleviated upon REST silencing (Fig. 3G). Moreover, the potential DNA binding sequence recognized by REST according to JASPAR database was found in our designed KIF15 promoter probe (Fig. 3H), and ChIP assay proved again the binding of REST at KIF15 promoter in GBM cells (Fig. 3I). Altogether, these results demonstrated that the abnormal up-regulation of KIF15 in GBM might be mediated by the transactivation of REST.

Since REST might be a potential regulator of KIF15 to be involved in the tumor-promoting role of KIF15 and no research focusing on the function of REST in GBM has been reported, we therefore next evaluated the intrinsic tumor-promoting role of REST in glioblastoma cells. Data retrieved from TCGA normal and GTEx database revealed that REST expression was significantly increased within GBM tissues compared to normal tissues (Supplementary Fig. 3A). Moreover, we acquired the recurrence and OS data from CGGA database. The expression of REST was higher in GBM with relapse than in those without relapse (Supplementary Fig. 3B) and the patients with high REST expression displayed much poorer prognosis (Supplementary Fig. 3C). Subsequently, higher REST expression in three cases of brain tumor tissues compared to normal tissues was shown in supplementary figure 3D. These results suggested that REST is positively associated with glioblastoma progression. Furthermore, we evaluated and found REST was highly expressed in different glioblastoma cells (Supplementary Fig. 3E), and ectopic REST expression increased KIF15 expression in GBM cells, while REST knockdown reduced KIF15 protein level (Supplementary Fig. 3F). In addition, cell viability, gap-closure and transwell assay showed that REST overexpression promotes GBM cell proliferation, migration and invasion, whereas its knockdown caused opposite effects (Supplementary Fig. 3G-I). Collectively, these data suggested that REST promotes the malignant phenotype of GBM* in vitro*, most likely, by targeting KIF15.

**Figure 4:**
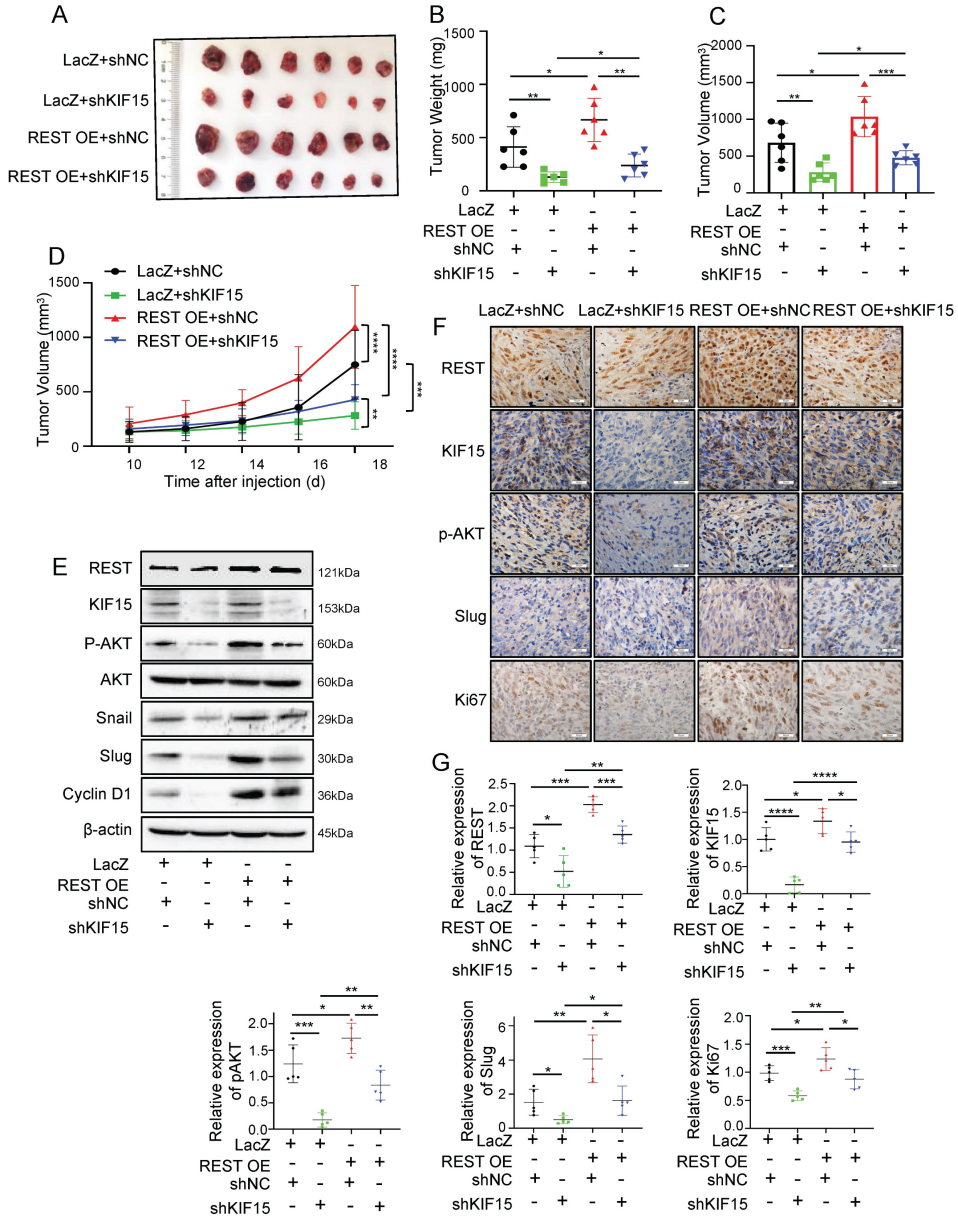
**REST promotes the growth of glioblastoma xenografts in mice partially by targeting KIF15.** (**A**) The formed tumors were stripped and photographed at the end of experiment. (**B-C**) The mice of each group were sacrificed, and the tumor weight and tumor volume was measured respectively. (**D**) The tumor diameter was measured at interval of 2 days after cell injection for 10 days and the tumor volume was calculated. (**E**) The expressions of REST, KIF15, p-AKT, AKT, Snail, Slug, Cyclin D1, and β-actin in tumor xenografts were measured by western blot. (**F**) REST, KIF15, p-AKT, Slug, and Ki67 in tumor xenografts were detected by immunohistochemistry staining and representative images were shown. Scale bars=50µm. Original magnification: ×40. (**G**) Statistical analysis for the expression of REST, KIF15, p-AKT, Slug and Ki67 in U87MG xenografts based on IHC staining, n=5. Data are presented as means±SD, Two-tailed unpaired Student's T-test. The level of significance was indicated by *P<0.05, **P<0.01, ***P<0.0001.

In order to explore whether REST promoted GBM progression dependent on its regulation on KIF15, we knocked down KIF15 expression in GBM cells on the basis of REST overexpression *in vitro*. Cell viability and transwell assay revealed that cell proliferation and invasion promoted by REST overexpression were partially reversed by KIF15 knockdown (Fig. 3J-L). To verify our *in vitro* data in an *in vivo* system, we further established U87MG cells with stable REST overexpression and/or KIF15 knockdown. We subcutaneously implanted such cells into NYG mice and evaluated tumor growth by measuring tumor size and weight. The results showed that REST overexpression partially alleviated tumor growth inhibition caused by KIF15 knockdown (Fig. 4A-D). Western blot and IHC staining of tumor tissues proved that the decreased expressions of p-AKT and EMT- and cell cycle-associated protein markers mediated by KIF15 knockdown were also rescued by REST overexpression (Fig. 4E, F). Together, all these data proved that KIF15 promotes glioblastoma progression under the targeted regulation of REST.

### P300 interacts with REST and its acetyltransferase activity was necessary for the transcriptional activation of KIF15 mediated by REST in glioblastoma

**Figure 5:**
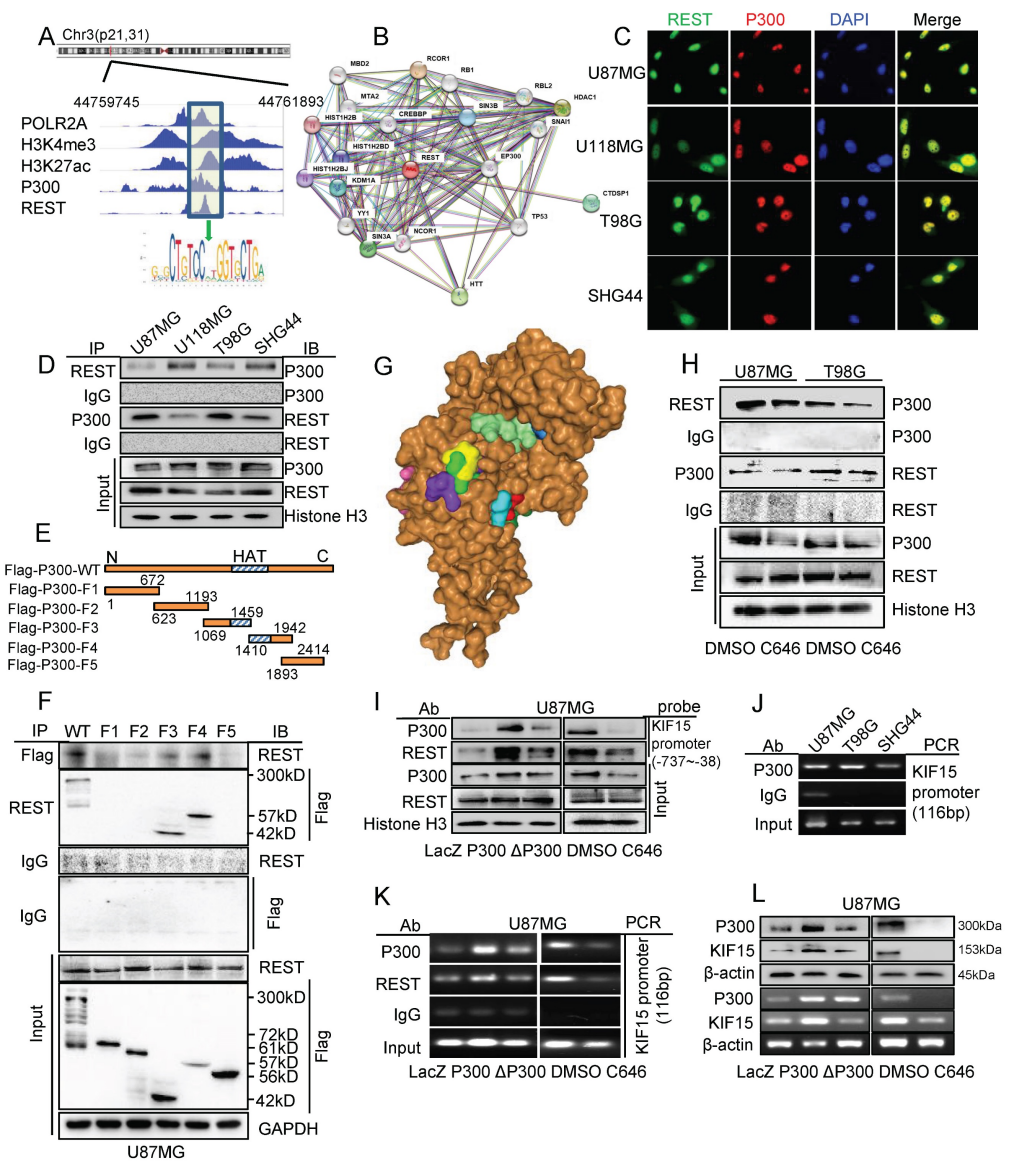
** P300 and its histone acetyltransferase activity were necessary for the transcriptional activation of KIF15 mediated by REST.** (**A**) The binding peaks of P300 and REST at the same region of KIF15 promoter in GBM cells from ChIP-seq data in Cistrome Browser. (**B**) The predicted interactions between REST and other proteins, including P300, by STRING databases. (**C**) Immunofluorescence assay was used to detect the colocalization of REST and P300 in different glioblastoma cells. (**D**) The interaction between REST and P300 in different GBM cells was detected by co-immunoprecipitation. (**E**) The schematic image of P300 plasmids with different domains was shown. (**F**) Co-IP assay was used to detect the interaction between REST and P300 with different truncates in U87MG cells. (**G**) The interaction of P300 HAT domain (brown) with the potential sites (colored) of REST residues (150-156 amino acids) was predicted by HDOCK software. (**H**) Co-IP assay was performed to detect the interaction between P300 and REST after U87MG and T98G cells were treated with DMSO or C646 for 24 h. (**I**) The binding of P300 and REST in KIF15 promoter was analyzed by DNA pulldown assay with KIF15 promoter probe (-737/-38) in U87MG cells with different treatment. (**J**) P300 antibody or IgG was used to immunoprecipitate KIF15 promoter fragment (-402 to -287) in different GBM cells by ChIP assay. (**K**) P300 antibody, REST antibody or IgG was used to immunoprecipitate KIF15 promoter fragment (-402 to -287) in U87MG cells after transfection with P300, ΔP300 plasmids or treated with C646. (**L**) The expressions of P300 and KIF15 in U87MG cells transfected with P300, ΔP300 plasmids or treated with C646 were detected by western blot and RT-PCR. The data represent the mean±SD of three independent experiments. ΔP300: P300 histone acetyltransferase domain delete mutation.

It has been reported that REST could silence gene activation via histone modification by forming regulatory complex with HDACs 25, we deduced the possible involvement of some other transcriptional activator or co-activator in the regulation of REST on KIF15 expression. We first searched the ChIP-seq data in Cistrome Data Browser and found that the KIF15 promoter region possesses highly activate state in human glioblastoma cells, which was indicated by the binding of H3K4me3 and H3K27ac. At the same time, the binding peak of P300 was also seen at KIF15 promoter region bound by REST (Fig. 5A). Considering the histone acetyltransferase activity of P300 and its function as a transcriptional co-activator, we next explored its synergy with REST in regulating KIF15 transcription. By using STRING database, we predicted the interaction between REST and P300 (Fig. 5B). Immunofluorescence staining showed the co-localization of P300 and REST in nucleus in different glioblastoma cells (Fig. 5C). Coimmunoprecipitation studies demonstrated clear associations between P300 and REST in GBM cells (Fig. 5D). To determine which domain of P300 is responsible for its interaction with REST, we constructed flag-tagged P300 plasmids with different deletion mutations in P300 domains (Fig. 5E). After transfecting these plasmids into U87MG cells, the isolated protein complexes were immunoprecipitated by Flag antibody or REST antibody. The result verified that REST interacted with P300 domain containing HAT activity (amino acids at sites 1069-1942) (Fig. 5F). In line with this, the prediction using HDOCK software also showed the binding of the specific amino acid sequence within the conformational space of REST with the HAT domain of P300 (Fig. 5G). To further explore the essential role of HAT activity of P300 in its interaction with REST, we performed another co-immunoprecipitation assay and found that P300 HAT inactivation reduced the interaction between P300 and REST in U87MG and T98G cells (Fig. 5H). DNA pulldown and ChIP assay was respectively performed in U87MG cells with overexpression of wild type P300 or HAT deletion mutant P300 (ΔP300) or P300 HAT activity inhibitor C646 treatment. The results showed that P300 overexpression induced more binding of P300 itself and REST at KIF15 promoter region, while ΔP300 overexpression caused no binding increase (Fig. 5I, K). By contrast, P300 HAT inhibitor C646 treatment reduced the binding of P300 itself and REST at KIF15 promoter region (Fig. 5I, K). ChIP assay in different GBM cells also proved that P300 could bind to the specific region of KIF15 promoter (Fig. 5J). Furthermore, the ChIP assay proved again the HAT activity of P300 was pivotal for P300 and REST binding with KIF15 promoter (Fig. 5K). Moreover, the overexpression of P300, but not ΔP300, augmented the expression of KIF15, while P300 HAT inhibition with C646 attenuated KIF15 expression (Fig. 5L). Overall, our results showed the essential role of P300 and its HAT activity in the anchoring of REST at KIF15 promoter region and, most likely, in the regulation of REST on KIF15 expression in glioblastoma progression.

### REST synergizes with P300 to co-regulate KIF15 expression and glioblastoma cell malignancy

**Figure 6:**
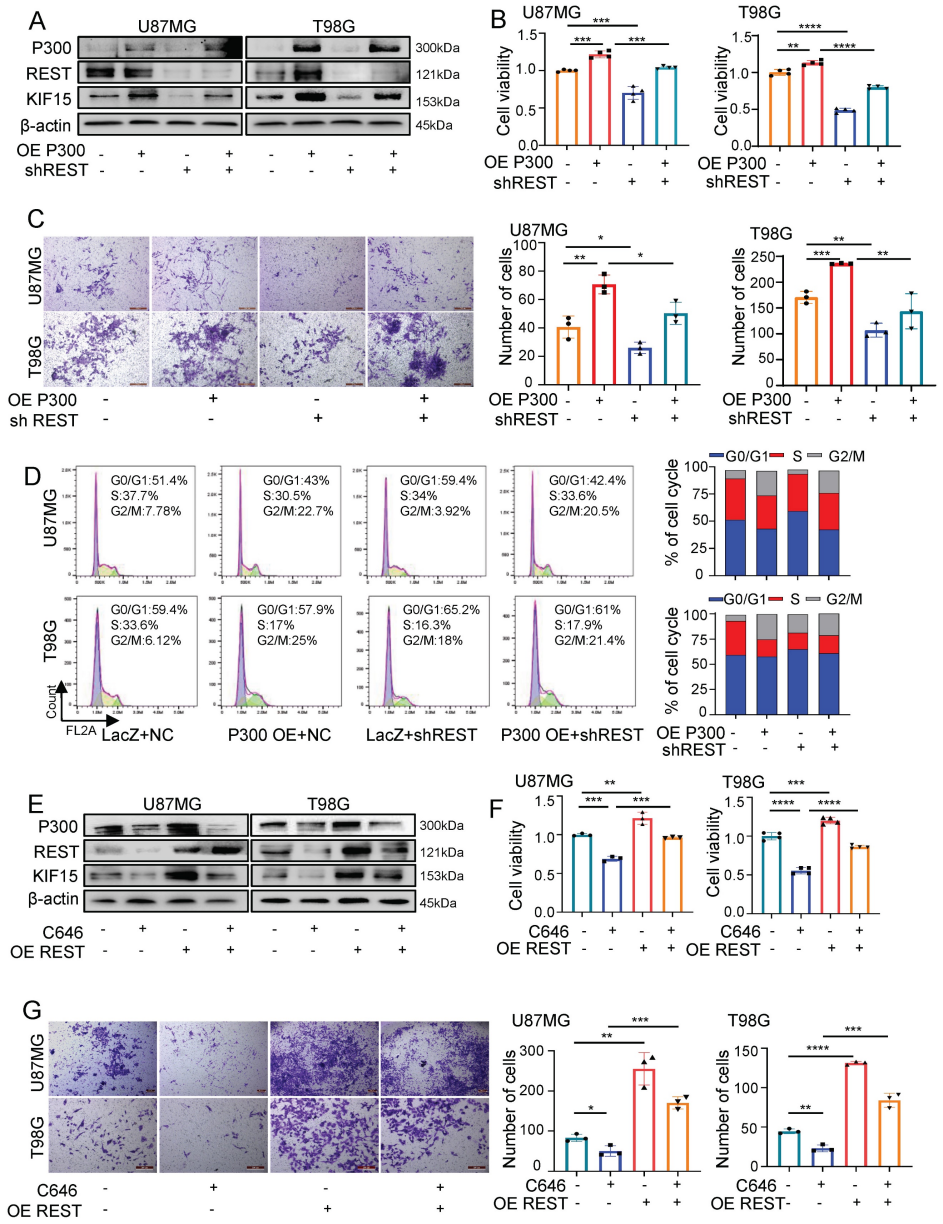
**REST synergizes with P300 to co-regulate KIF15 expression and glioblastoma cell malignancy.** (**A**) The expressions of P300, REST and KIF15 were detected in U87MG and T98G cells with P300 overexpression and/or REST knockdown. (**B-D**) MTT (**B**), transwell (**C**), and cell cycle assay (**D**) was performed respectively in U87MG and T98G cells with P300 overexpression and/or REST knockdown. The corresponding quantified analysis was also performed. (**E**) The expressions of P300, REST and KIF15 were detected in U87MG and T98G cells with C646 treatment (10µM) and/or REST overexpression. (**F-G**) MTT assay (**F**) and transwell assay (**G**) was performed in U87MG and T98G cells treated with C646 and/or REST overexpression. The data represent the mean±SD of three independent experiments, and the level of significance was indicated by *P<0.05, **P<0.01, ***P<0.001.

Prompted by above findings, we next explored the possibility that REST associates with P300 to co-regulate glioblastoma cell malignancy by synergistically targeting KIF15. For this purpose, we first overexpressed P300 and simultaneously knocked down REST in U87MG and T98G cells. As shown in figure 6A, the up-regulated expression of KIF15 mediated by P300 overexpression was alleviated by REST silencing. Not only that, cell viability, transwell and FACS assays revealed that cell proliferation, invasion and cell cycle progression promoted by P300 overexpression were reversed by REST knockdown (Fig. 6B-D). Conversely, P300 HAT inactivity by C646 treatment suppressed GBM cell proliferation and invasion while REST overexpression resulted in the reversal of such suppression (Fig. 6E-G).

To further clarify the clinical correlation between pairwise of REST, P300 and KIF15 in GBM, we investigated the clinical significance of P300 in GBM patients. P300 was found to be highly expressed in GBM compared to normal brain tissues based on TCGA normal and GTEx database, although the difference has no statistical significance (Supplementary Fig. 4A). IHC staining showed that the expression of P300 in GBM is higher than that in normal brain tissues in the data downloaded from Human Protein Atlas (Supplementary Fig. 4B). According to the data from GEPIA database, P300 expression could not predict GBM patients' survival (Supplementary Fig. 4C). However, P300 expression is moderately correlated with KIF15 expression and highly correlated with REST expression in GBM patients (R=0.36 and 0.57 respectively) (Supplementary Fig. 4D, E). More importantly, the simultaneous hyper-expression of P300/REST/KIF15 could effectively separate GBM tissue from normal brain tissues (Supplementary Fig. 4F) and high expression of these three simultaneously predict poor prognosis in glioblastoma patients (Supplementary Fig. 4F, G). These results, from another perspective, proved again the synergy of REST and P300 in promoting KIF15 expression and GBM malignancy and their potential in clinical diagnosis and outcome prediction.

### C646 treatment improves the therapeutic efficacy of palbociclib in GBM xenograft models

**Figure 7:**
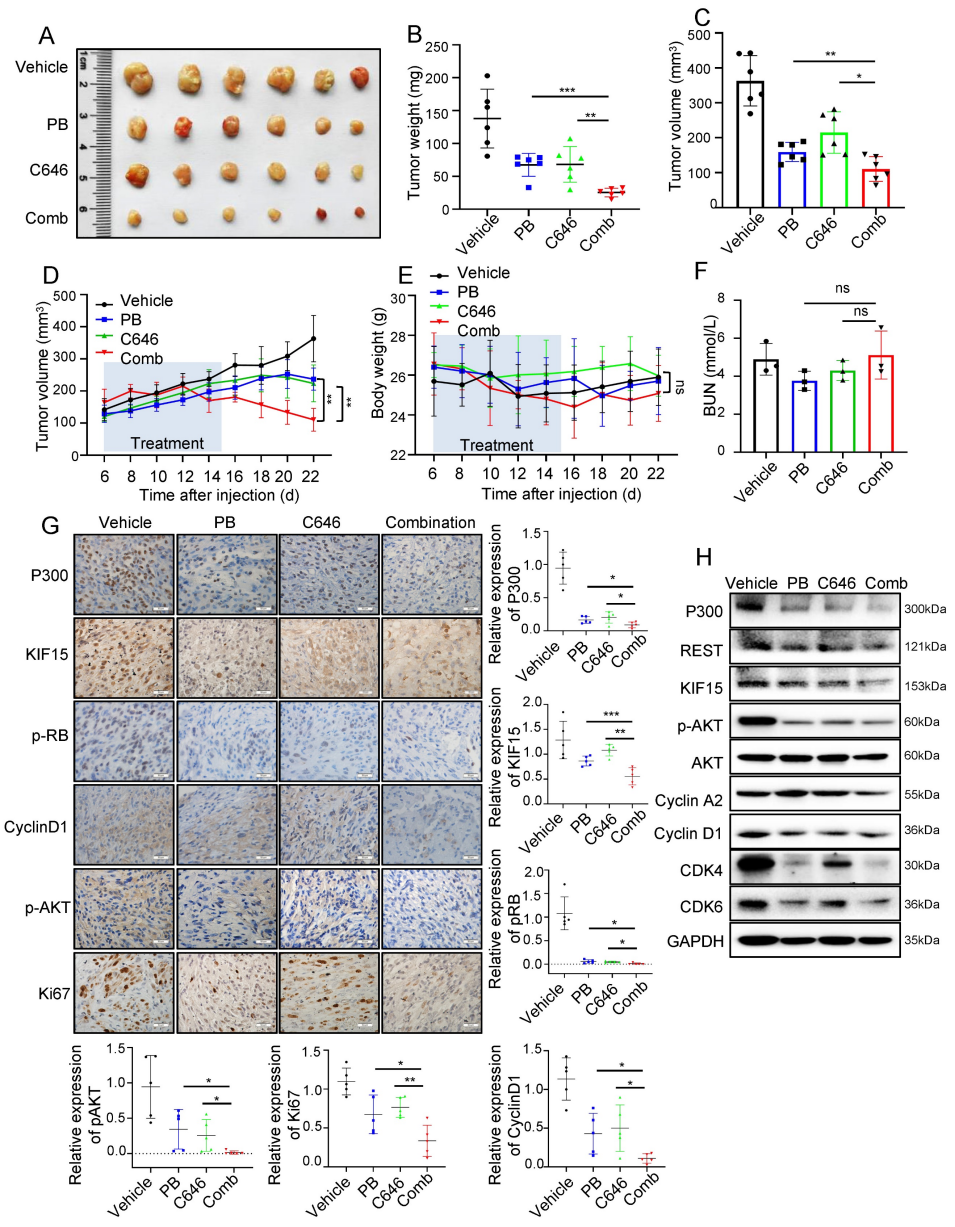
**C646 combined with Palbociclib produced synergistic anti-tumor effect in glioblastoma xenograft model.** (**A**) The representative images of tumor xenografts from mice treated with indicated agents. (**B-C**) Tumor weight (**B**) and volume (**C**) was respectively measured or calculated after mice were sacrificed. (**D**) Tumor volumes of NYG mice bearing U87MG xenografts were measured at the 6th day after cell injection every two days. (**E**) The changes in body weight for mice bearing xenografts were recorded during the whole treatment process. (**F**) Blood Urea Nitrogen (BUN) levels of mice in each group were measured by the BUN kit (n=6). (**G**) The expressions of P300, KIF15, p-RB, CyclinD1, p-AKT, and Ki67 were analyzed by immunohistochemical staining in U87MG xenografts after receiving different treatments (n=5) and the representative images and the corresponding quantified analysis were also shown. Scale bars, 50µm. Data are presented as means±SD, Two-tailed unpaired Student's T-test. (**H**) The expressions of P300, REST, KIF15, p-AKT, Cyclin A2, Cyclin D1, CDK4, CDK6 and GAPDH from tumor tissue lysates were analyzed by Western blot. The data represent the mean±SD of three independent experiments, and the level of significance was indicated by *P<0.05, **P<0.01, ***P<0.001. PB: Palbociclib; Comb: Combination.

Since REST regulates KIF15 expression and subsequent cell cycle progression dependent on P300 and its HAT activity in GBM cells, we reasonably supposed HAT inactivity of P300 might sensitize GBM to cell cycle inhibitor treatment. We next determined whether the combination of C646 and Palbociclib displays better therapeutic efficacy than Palbociclib alone in mouse xenograft models. Mice bearing U87MG xenografts were treated with Vehicle, Palbociclib, C646 or their combination at the 6th days after cell injection for another 10 days and tumor growth trend was recorded and analyzed. Compared to monotherapy, combination therapy more significantly suppressed tumor growth (Fig. 7A-D), while mice weight loss and overt toxicity were not found (Fig. 7E, F). Consistently, the expressions of cell cycle-associated and proliferation-associated protein markers in tumor tissues were obviously down-regulated upon the combined treatment when compared to the single treatment group (Fig. 7G, H). These data indicated that the combination of Palbociclib and C646 might be an effective therapeutic strategy for glioblastoma, further implying that P300 HAT inactivation might be considered to be employed to sensitize GBM cell to cell cycle blockade.

## Discussion

**Figure 8:**
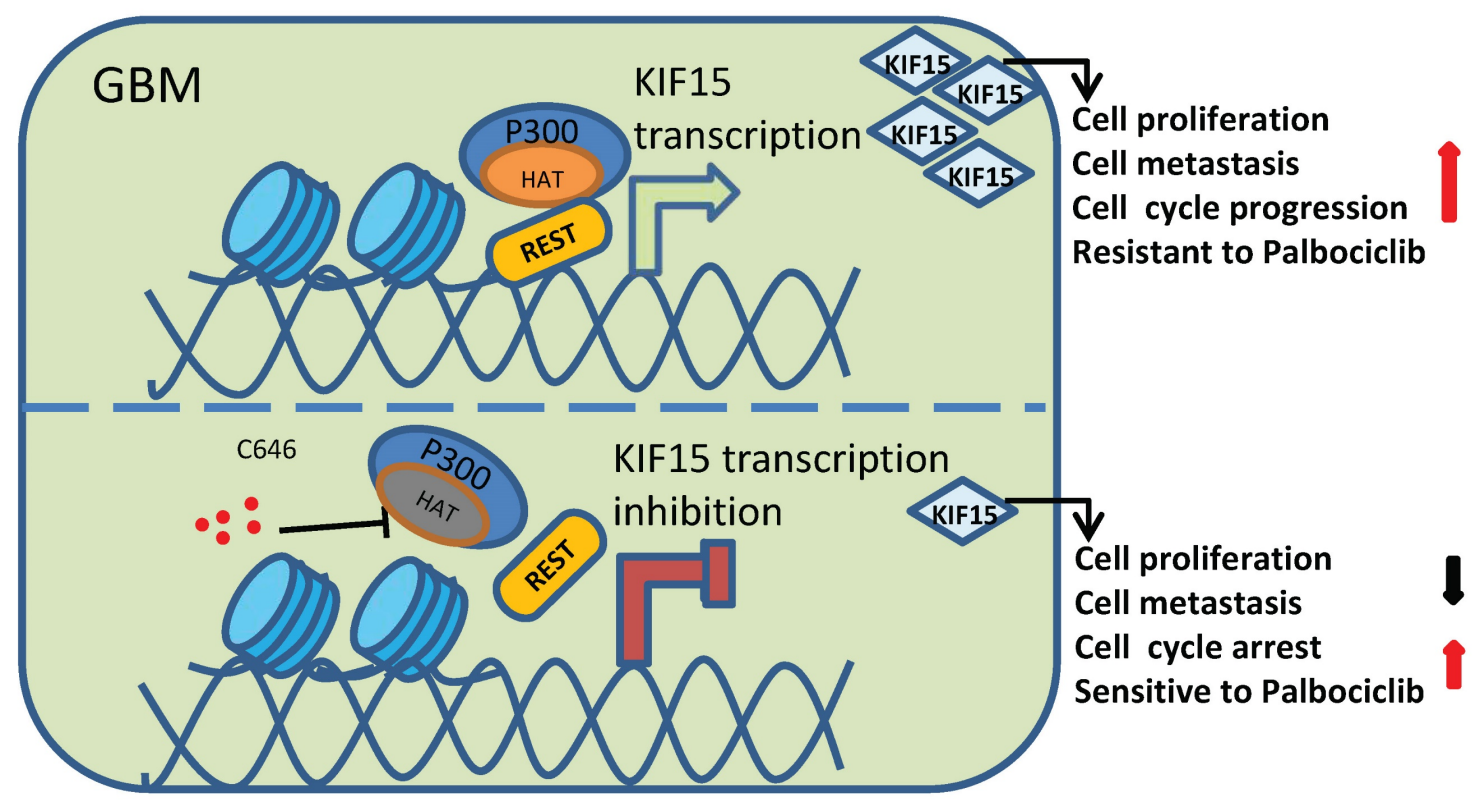
** Schematic diagram of KIF15 regulate mechanism in GBM.** We demonstrated that REST recruits P300 to co-anchor at the promoter region of KIF15 in GBM cells to synergistically regulate KIF15 transcription and subsequent tumor progression, during which the HAT activity of P300 has been proved necessity to potentiate KIF15 promoter activity.

Among different types of brain tumors, glioblastoma is a WHO grade IV glioma characterized by property of high invasiveness, extensive metastasis, and rapid angiogenesis. Combined with genetic heterogeneity and protection by the blood brain barrier, great treatment challenges have been posed to GBM. More than that, the rapid DNA repair and self-renewing capacities of GBM cells lead to resistance production to all common treatment patterns, including temozolomide (TMZ) treatment, which has been approved by the FDA for patients with GBM and astrocytomas 31. Thus, it's urgent to find new biological and molecular targets in GBM progression and to develop innovative treatment strategy based on this. Our study demonstrated that KIF15 promotes GBM cell proliferation, metastasis, cell cycle progression and resistance to Palbociclib. Mechanistically, REST interacts with P300 histone acetyltransferase (HAT) domain to co-regulate KIF15 expression. Inhibition of P300 HAT activity with C646 leads to KIF15 transcription inhibition and the alleviated KIF15 expression, thereby further resulting in the decreased cell proliferation and metastasis and the increased cell cycle arrest and sensitivity to Palbociclib (Figure 8). Thus, P300/REST/KIF15 signaling axis is expected to be served as a cascade of candidate therapeutic targets in anti-GBM.

A linkage between REST and KIF15 in glioblastoma cancer has been built in our study based upon several new findings. First, REST has been found to be recruited to the KIF15 promoter and activate KIF15 transcription in GBM cells, which provides a deep understanding in the regulatory mechanism of KIF15's abnormal activation in glioblastoma. Second, REST has been identified as an oncogene in glioblastoma in our study, which is consist with the finding that REST plays tumor-promoting roles in nervous system cancer 32,33, although in non-nervous system cancer, it has been implicated as a transcription repressor to play diverse roles in multiple cellular processes 34, 35. As evidenced by the results that KIF15 knockdown reversed REST overexpression-caused GBM cell survival promotion *in vitro* and *in vivo*, it is conclusive that REST drives GBM progression at least partially via its targeted regulation on KIF15. In other words, KIF15 plays an integral role in the tumor-promoting function of REST in GBM, thereby indirectly proving the importance of KIF15 in GBM development.

Given the function of P300 as transcription co-activator and the appearance of its binding peaks at KIF15 promoter region in GBM cells, which has been proved to be bound to by REST, we deduced the possibility of P300 interacting with REST to co-regulate KIF15 transcription. Combined with the previous studies showing that REST binds to the RE1 motif within target genes and recruits Sin3A/B and coREST to repress gene expression 25, 36 and P300 interacts with RBBP4 to form complex that controls critical survival genes and respond for TMZ resistance in glioblastoma 37, it is more reasonable to assume that REST synergizes with P300 to co-regulate GBM progression by targeted activation of KIF15 transcription. In line with this, we identified the existence of P300/REST axis at KIF15 promoter region to co-modulate the expression of KIF15, and further to co-promote GBM cell proliferation and metastasis. Moreover, the interaction between P300 HAT domain and REST has been verified in our results (Fig. 5E-G), and the binding of REST at KIF15 promoter was dependent on the HAT activity of P300, which is reflected by the facts that HAT function loss mutant of P300 or its HAT inhibitor could not influence or decrease the binding of REST at KIF15 promoter. We thus speculated that REST recruits P300 to the promoter of KIF15 gene to initiate KIF15's transcription under the acetylation of itself mediated by P300. Considering that the binding of REST on KIF15 promoter region was accompanied by H3K4 methylation and H3K27 acetylation predicted in Wash U epigenetic database (Fig. 5A), the possibility that acetylation of histones at this fragment is also mediated by P300 is not excluded. If so, very possibly, histone and REST share different sites within P300 HAT domain. Besides, some other transcription activators or coactivators might also be needed to initiate KIF15's transcription together with REST and P300 in GBM cells. All these points deserve to be further investigated and clarified.

To date, clinical trial approaches for GBM treatment focusing on glioblastoma-intrinsic targets including tyrosine receptor kinases 38-40, cell cycle control 41 and apoptosis induction 42. But the effects were disappointing possibly because of the inadequacy of single pathway blocking or production of therapeutic resistance. The same situation has occurred in the clinical trials of cell cycle inhibitors, especially CDK inhibitors, including Palbociclib, Ribociclib, and Abemaciclib, in GBM patients, although they have been approved by the FDA for the treatment of advanced breast cancer 43 and Palbociclib was found to efficiently cross the blood-brain barrier and proved highly effective in suppressing the growth of intracranial GBM xenograft tumors 44. Thus, the adjuvant or combination treatment strategies for cell cycle blockade should be explored and proposed. Based on our finding that KIF15 plays a tumor-promoting role in glioblastoma by accelerating cell cycle progression and P300 HAT activity contributes to the abnormal up-regulation of KIF15 mediated by REST in GBM, we explored and proved the potential of Palbociclib combined with P300 inhibitor (C646) in anti-GBM, which is consistent with our clinical finding that P300/REST/KIF15 axis is positively correlated with the malignant progression of glioblastoma patients. For the incurable glioblastoma, these findings will hopefully lead to its improved treatment. Of note, KIF15-IN-1, a commercially available small molecule inhibitor on KIF15 motility 45, has been reported to be used in castration-resistant prostate cancer (CRPC) treatment 46. Considering the pivotal role of KIF15 in glioblastoma progression proved by us, whether KIF15-IN-1 could be used in anti-glioblastoma alone or in combination with other treatments, including chemotherapy or cell cycle suppression, should also be further investigated. From the current research, our study at least provides a theoretical basis for the usage of KIF15-IN-1 in glioblastoma treatment.

Our study currently focuses on the comprehensive exploration about the upstream regulatory mechanisms of KIF15 regarding to its promotion in GBM progression, although MTT assay in Fig. S1 preliminarily indicated that, both Erk/AKT and cell cycle pathway were responsible for the tumor-promoting role of KIF15 as the possible key downstream signaling pathways. KIF15 knockdown was found to cause cell cycle blockade and sensitize GBM cells to Palbociclib treatment. Combined with the known role of KIF15 in cell mitosis and the potential regulation of cell cycle progression by Erk/AKT pathway, we cannot rule out the main contribution of the cell cycle in the GBM promotion process mediated by KIF15. To further uncover the downstream effective mechanisms of KIF15 in GBM development, more explorations, including RNA-sequencing study, are needed and should be conducted in our future study.

## Supplementary Material

Supplementary figures.

## Abbreviations

GBM: Glioblastoma
KIF15: Kinesin family member 15
REST: Repressor element-1(RE1) silencing transcription factor
NRSF: Neuron-restrictive silencer factor
KPB: Kif1-binding protein
EMT: Epithelial mesenchymal transition
ChIP: Chromatin immunoprecipitation
Co-IP: Co-immunoprecipitation
IF: Immunofluorescence
IHC: Immunohistochemistry staining
OS: Overall survival
HDACs: Histone deacetylases
P300: E1A binding protein P300, EP300
ΔP300: P300 HAT deletion mutant
HAT: Histone acetyltransferases
PB: Palbociclib
TMZ: Temozolomide
TCGA: The cancer genome atlas
GEO: Gene expression omnibus

## References

[1] Thomas DL (2023). 2021 updates to the World Health Organization classification of adult-type and pediatric-type diffuse gliomas: a clinical practice review. Chinese clinical oncology.

[2] Veliz I, Loo Y, Castillo O, Karachaliou N, Nigro O, Rosell R (2015). Advances and challenges in the molecular biology and treatment of glioblastoma-is there any hope for the future?. Annals of translational medicine.

[3] Ratliff M, Karimian-Jazi K, Hoffmann DC, Rauschenbach L, Simon M, Hai L (2023). Individual glioblastoma cells harbor both proliferative and invasive capabilities during tumor progression. Neuro-oncology.

[4] Nehama D, Woodell AS, Maingi SM, Hingtgen SD, Dotti G (2023). Cell-based therapies for glioblastoma: Promising tools against tumor heterogeneity. Neuro-oncology.

[5] Pellerino A, Bruno F, Soffietti R, Rudà R (2023). Antiangiogenic Therapy for Malignant Brain Tumors: Does It Still Matter?. Current oncology reports.

[6] Mann BJ, Balchand SK, Wadsworth P (2017). Regulation of Kif15 localization and motility by the C-terminus of TPX2 and microtubule dynamics. Molecular biology of the cell.

[7] Buster DW, Baird DH, Yu W, Solowska JM, Chauvière M, Mazurek A (2003). Expression of the mitotic kinesin Kif15 in postmitotic neurons: implications for neuronal migration and development. Journal of neurocytology.

[8] Brouwers N, Mallol Martinez N, Vernos I (2017). Role of Kif15 and its novel mitotic partner KBP in K-fiber dynamics and chromosome alignment. PloS one.

[9] Quan G, Xu J, Wang J, Liu X, Xu J, Jiang J (2023). KIF15 is essential for USP10-mediated PGK1 deubiquitination during the glycolysis of pancreatic cancer. Cell death & disease.

[10] Bi H, Hou X, Shen Q, Liu Z, Zhu X, Ma L (2023). Knockdown of KIF15 suppresses proliferation of prostate cancer cells and induces apoptosis through PI3K/Akt signaling pathway. Cell death discovery.

[11] Shi D, Wang J, Deng Q, Kong X, Dong Y, Yang Y (2023). KIF15 knockdown inhibits colorectal cancer proliferation and migration through affecting the ubiquitination modification of NRAS. American journal of cancer research.

[12] Cai Y, Lai Q, Zhang X, Zhang Y, Zhang M, Gu S (2023). Kinesin superfamily member 15 knockdown inhibits cell proliferation, migration, and invasion in nasopharyngeal carcinoma. The Korean journal of physiology & pharmacology: official journal of the Korean Physiological Society and the Korean Society of Pharmacology.

[13] Qiao Y, Chen J, Ma C, Liu Y, Li P, Wang Y (2018). Increased KIF15 Expression Predicts a Poor Prognosis in Patients with Lung Adenocarcinoma. Cellular physiology and biochemistry: international journal of experimental cellular physiology, biochemistry, and pharmacology.

[14] Gupta SK, Gressens P, Mani S (2009). NRSF downregulation induces neuronal differentiation in mouse embryonic stem cells. Differentiation; research in biological diversity.

[15] Tomasoni R, Negrini S, Fiordaliso S, Klajn A, Tkatch T, Mondino A (2011). A signaling loop of REST, TSC2 and β-catenin governs proliferation and function of PC12 neural cells. Journal of cell science.

[16] Antoniotti S, Ruffinatti FA, Torriano S, Luganini A, D'Alessandro R, Lovisolo D (2016). REST levels affect the functional expression of voltage dependent calcium channels and the migratory activity in immortalized GnRH neurons. Neuroscience letters.

[17] Xue WJ, He CF, Zhou RY, Xu XD, Xiang LX, Wang JT (2022). High glucose and palmitic acid induces neuronal senescence by NRSF/REST elevation and the subsequent mTOR-related autophagy suppression. Molecular brain.

[18] Lu T, Aron L, Zullo J, Pan Y, Kim H, Chen Y (2014). REST and stress resistance in ageing and Alzheimer's disease. Nature.

[19] Kawamura M, Sato S, Matsumoto G, Fukuda T, Shiba-Fukushima K, Noda S (2019). Loss of nuclear REST/NRSF in aged-dopaminergic neurons in Parkinson's disease patients. Neuroscience letters.

[20] He CF, Xue WJ, Xu XD, Wang JT, Wang XR, Feng Y (2022). Knockdown of NRSF Alleviates Ischemic Brain Injury and Microvasculature Defects in Diabetic MCAO Mice. Frontiers in neurology.

[21] Nishimura E, Sasaki K, Maruyama K, Tsukada T, Yamaguchi K (1996). Decrease in neuron-restrictive silencer factor (NRSF) mRNA levels during differentiation of cultured neuroblastoma cells. Neuroscience letters.

[22] Dobson THW, Tao RH, Swaminathan J, Maegawa S, Shaik S, Bravo-Alegria J (2019). Transcriptional repressor REST drives lineage stage-specific chromatin compaction at Ptch1 and increases AKT activation in a mouse model of medulloblastoma. Science signaling.

[23] Callegari K, Maegawa S, Bravo-Alegria J, Gopalakrishnan V (2018). Pharmacological inhibition of LSD1 activity blocks REST-dependent medulloblastoma cell migration. Cell communication and signaling: CCS.

[24] Shaik S, Maegawa S, Haltom AR, Wang F, Xiao X, Dobson T (2021). REST promotes ETS1-dependent vascular growth in medulloblastoma. Molecular oncology.

[25] Roopra A, Sharling L, Wood IC, Briggs T, Bachfischer U, Paquette AJ (2000). Transcriptional repression by neuron-restrictive silencer factor is mediated via the Sin3-histone deacetylase complex. Molecular and cellular biology.

[26] Ding N, Tomomori-Sato C, Sato S, Conaway RC, Conaway JW, Boyer TG (2009). MED19 and MED26 are synergistic functional targets of the RE1 silencing transcription factor in epigenetic silencing of neuronal gene expression. The Journal of biological chemistry.

[27] Medellin B, Yang W, Konduri S, Dong J, Irani S, Wu H (2022). Targeted Covalent Inhibition of Small CTD Phosphatase 1 to Promote the Degradation of the REST Transcription Factor in Human Cells. Journal of medicinal chemistry.

[28] Zeng Q, Wang K, Zhao Y, Ma Q, Chen Z, Huang W (2023). Effects of the Acetyltransferase p300 on Tumour Regulation from the Novel Perspective of Posttranslational Protein Modification. Biomolecules.

[29] Kalkhoven E (2004). CBP and p300: HATs for different occasions. Biochemical pharmacology.

[30] Haery L, Mussakhan S, Waxman DJ, Gilmore TD (2016). Evidence for an oncogenic modifier role for mutant histone acetyltransferases in diffuse large B-cell lymphoma. Leukemia & lymphoma.

[31] Lee SY (2016). Temozolomide resistance in glioblastoma multiforme. Genes & diseases.

[32] Jin L, Liu Y, Wu Y, Huang Y, Zhang D (2023). REST Is Not Resting: REST/NRSF in Health and Disease. Biomolecules.

[33] Fuller GN, Su X, Price RE, Cohen ZR, Lang FF, Sawaya R (2005). Many human medulloblastoma tumors overexpress repressor element-1 silencing transcription (REST)/neuron-restrictive silencer factor, which can be functionally countered by REST-VP16. Molecular cancer therapeutics.

[34] Westbrook TF, Martin ES, Schlabach MR, Leng Y, Liang AC, Feng B (2005). A genetic screen for candidate tumor suppressors identifies REST. Cell.

[35] Lv H, Pan G, Zheng G, Wu X, Ren H, Liu Y (2010). Expression and functions of the repressor element 1 (RE-1)-silencing transcription factor (REST) in breast cancer. Journal of cellular biochemistry.

[36] Ooi L, Wood IC (2007). Chromatin crosstalk in development and disease: lessons from REST. Nature reviews Genetics.

[37] Mladek AC, Yan H, Tian S, Decker PA, Burgenske DM, Bakken K (2022). RBBP4-p300 axis modulates expression of genes essential for cell survival and is a potential target for therapy in glioblastoma. Neuro-oncology.

[38] Weller M, Butowski N, Tran DD, Recht LD, Lim M, Hirte H (2017). Rindopepimut with temozolomide for patients with newly diagnosed, EGFRvIII-expressing glioblastoma (ACT IV): a randomised, double-blind, international phase 3 trial. The Lancet Oncology.

[39] van den Bent MJ, Gao Y, Kerkhof M, Kros JM, Gorlia T, van Zwieten K (2015). Changes in the EGFR amplification and EGFRvIII expression between paired primary and recurrent glioblastomas. Neuro-oncology.

[40] Wen PY, Touat M, Alexander BM, Mellinghoff IK, Ramkissoon S, McCluskey CS (2019). Buparlisib in Patients With Recurrent Glioblastoma Harboring Phosphatidylinositol 3-Kinase Pathway Activation: An Open-Label, Multicenter, Multi-Arm, Phase II Trial. Journal of clinical oncology: official journal of the American Society of Clinical Oncology.

[41] Taylor JW, Parikh M, Phillips JJ, James CD, Molinaro AM, Butowski NA (2018). Phase-2 trial of palbociclib in adult patients with recurrent RB1-positive glioblastoma. Journal of neuro-oncology.

[42] Vassilev LT, Vu BT, Graves B, Carvajal D, Podlaski F, Filipovic Z (2004). In vivo activation of the p53 pathway by small-molecule antagonists of MDM2. Science (New York, NY).

[43] Cejuela M, Gil-Torralvo A, Castilla M, Domínguez-Cejudo M, Falcón A, Benavent M (2023). Abemaciclib, Palbociclib, and Ribociclib in Real-World Data: A Direct Comparison of First-Line Treatment for Endocrine-Receptor-Positive Metastatic Breast Cancer. International journal of molecular sciences.

[44] Michaud K, Solomon DA, Oermann E, Kim JS, Zhong WZ, Prados MD (2010). Pharmacologic inhibition of cyclin-dependent kinases 4 and 6 arrests the growth of glioblastoma multiforme intracranial xenografts. Cancer research.

[45] Milic B, Chakraborty A, Han K, Bassik MC, Block SM (2018). KIF15 nanomechanics and kinesin inhibitors, with implications for cancer chemotherapeutics. Proceedings of the National Academy of Sciences of the United States of America.

[46] Gao L, Zhang W, Zhang J, Liu J, Sun F, Liu H (2021). KIF15-Mediated Stabilization of AR and AR-V7 Contributes to Enzalutamide Resistance in Prostate Cancer. Cancer research.

