# Supplementary material for: KIF15 promotes human glioblastoma progression under the synergistic transactivation of REST and P300
Source: Int J Biol Sci. 2024 Sep 23;20(13):5127–44. doi: 10.7150/ijbs.98668 (PMC11488581; doi:10.7150/ijbs.98668)
Supplement: Supplementary file 1 — Supplementary figures. [file ijbsv20p5127s1.pdf]

Supplementary figure legends

Figure S1. **KIF15 is highly expressed in glioblastoma cells and promotes cancer cell growth by targeting Erk/Akt pathway.** (**A**) The expression and localization of KIF15 in different glioblastoma cell lines were observed by immunofluorescence assay. (**B-D**) U87MG and T98G cells were treated with U0126 at 10 μM (**B**), LY294002 at 10 μM (**C**), or Palbociclib at 20 μM (**D**) for 4 hours, and then were transfected with control siRNAs or KIF15 siRNAs. After 48 h, cell viability was measured by MTT assay. (**E-G**) U87MG and T98G cells were transfected with KIF15 overexpression plasmids or empty vector（EV）for 48 h, and then were treated with U0126(**E**), LY294002(**F**) or Palbociclib(**G**) for 24 h. Cell viability was measured by MTT assay. The data represent the mean±SD of three independent experiments, and the level of significance was indicated by *P<0.05,**P<0.01, ***P<0.001.

Figure S2. **The expression correlation of KIF15 and cell cycle-related genes in glioblastoma are analyzed based on databases.** (**A**) The expression correlation data for individual cell cycle-related genes (y-axis) versus KIF15expression (x-axis) extracted for TCGA GBM. Each dot represents a tumor case (n=528 patients). The statistical significance of the correlation was determined using the Pearson's correlation coefficient. A red linear regression line is shown in each plot. (**B**) Visualization result showed the correlation between every two gens by R value.

Figure S3. **High REST expression predicts glioblastoma patient's poor prognosis and promotes the malignant phenotype of glioblastoma cells.** (**A**) REST expression is highly upregulated in glioblastoma compared to normal brain tissues in TCGA and GTEx databases. (**B-C**) REST expression is upregulated in recurrent glioblastoma compared to primary tumors and its high expression predicts poor prognosis of glioma patients in CGGA database. (**D**) REST expression in three cases of brain tumors compared to normal tissues and the scatter plot was displayed according to the quantified analysis of the IHC score. (**E**) The expression of REST was detected in different glioblastoma cells. (**F**) The expressions of REST and KIF15 were detected in U87MG and T98G cells after REST was overexpressed or knocked down. (**G**) Cell viability was measured by MTT assay after REST overexpression or knockdown at 48 hours. (**H-I**) Transwell assay (**H**) and gap closure assay (**I**) were performed respectively in U87MG and T98G cells after REST overexpression or knockdown at 24 hours or 48 hours respectively. The data represent the mean $\pm$ SD of three independent experiments, and the level of significance was indicated by $^*P<0.05$, $^{**}P<0.01$, $^{***}P<0.001$.

Figure S4. P300 expression is positively correlated with REST or KIF15 expression in glioblastoma tissues and the simultaneous high expression of these three proteins predicts poor prognosis of glioblastoma patients. (**A**) P300 expression is up-regulated in glioblastoma compared to normal brain tissues in TCGA and GTEx databases. (**B**) P300 expression in three cases of brain tumors compared to normal tissues and the corresponding quantified analysis according to the IHC score. (**C**) The survival curve of GBM patients with P300 high or low expression was downloaded according to GEPIA databases. (**D**) The correlations between P300 and KIF15 expression in glioblastoma patients from GEPIA database. (**E**) The correlations between P300 and REST expression in glioblastoma patients from GEPIA database. (**F**) The combinational expression of P300/REST/KIF15 could separate tumor tissues from other normal tissues in brain by dimensionality reduction analysis in GEPIA databases. (**G**) The overall survival of glioblastoma patients with high or low expression of P300/REST/KIF15 was analyzed in GEIPA database.

Figure S1

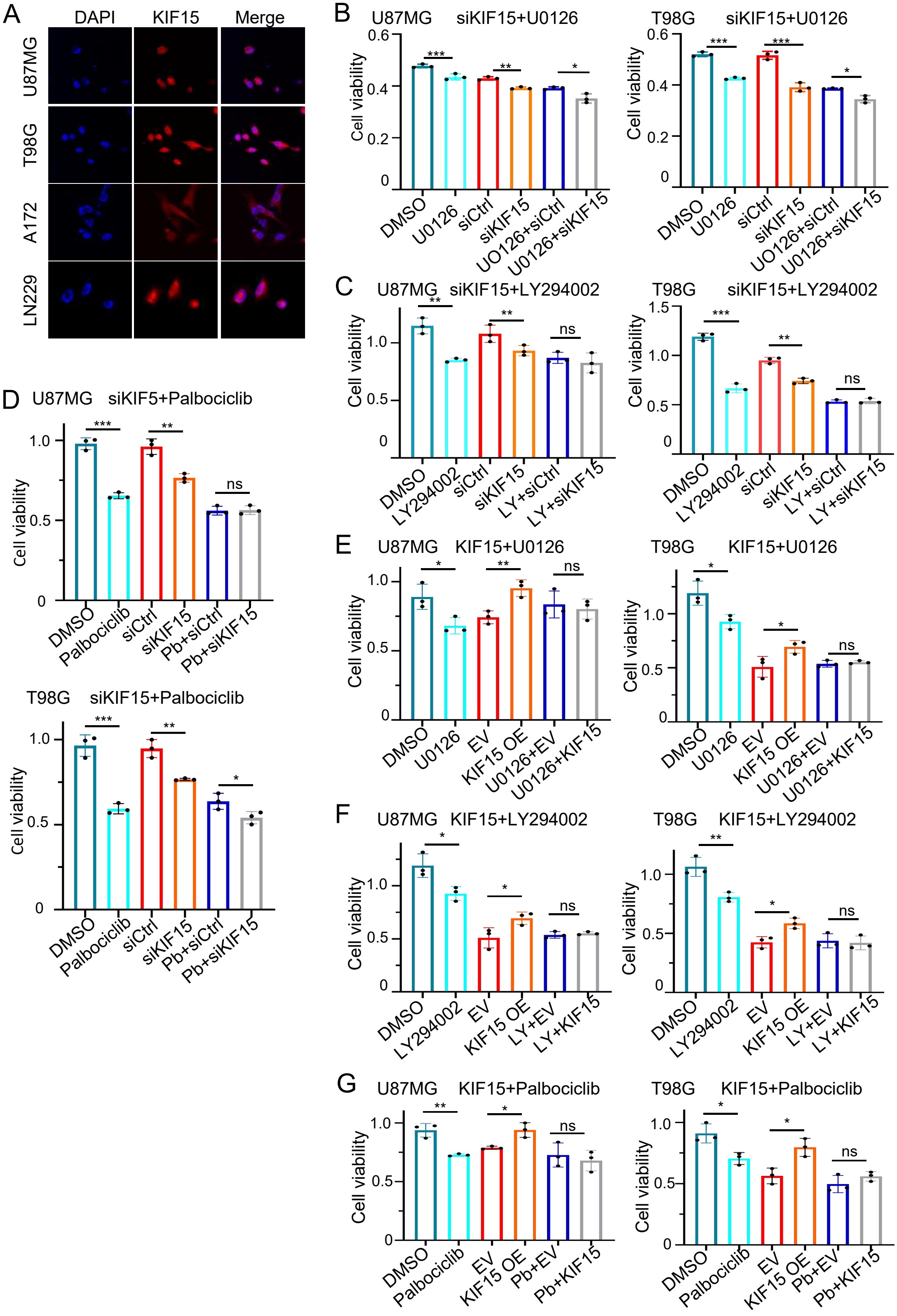
A
DAPI
KIF15
Merge
U87MG
T98G
A172
LN229
B
U87MG siKIF15+U0126
T98G siKIF15+U0126
Cell viability
DMSO
U0126
siCtrl
siKIF15
UO126+siCtrl
U0126+siKIF15
C
U87MG siKIF15+LY294002
T98G siKIF15+LY294002
LY294002
LY+siCtrl
LY+siKIF15
D
U87MG siKIF5+Palbociclib
T98G siKIF15+Palbociclib
Palbociclib
Pb+siCtrl
Pb+siKIF15
E
U87MG KIF15+U0126
T98G KIF15+U0126
EV
KIF15 OE
U0126+EV
U0126+KIF15
F
U87MG KIF15+LY294002
T98G KIF15+LY294002
LY+EV
LY+KIF15
G
U87MG KIF15+Palbociclib
T98G KIF15+Palbociclib
Pb+EV
Pb+KIF15
ns

Figure S2

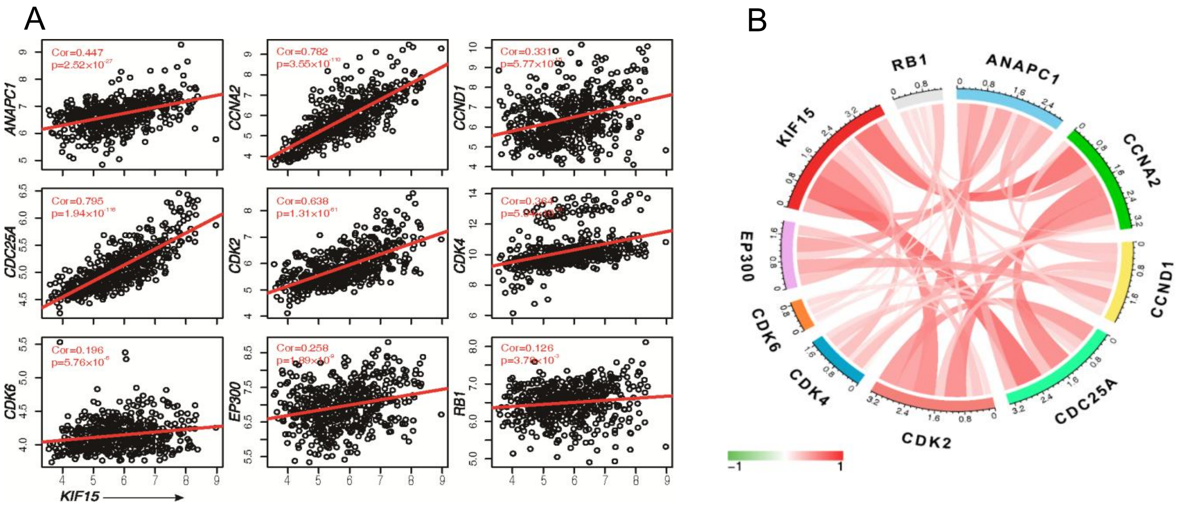
A
Cor=0.447
p=2.52×10⁻²⁷
ANAPC1
Cor=0.782
CCNA2
Cor=0.331
CCND1
Cor=0.795
CDC25A
Cor=0.638
CDK2
CDK4
Cor=0.196
CDK6
Cor=0.258
EP300
Cor=0.126
RB1
KIF15
B
RB1
ANAPC1
CCNA2
CCND1
CDC25A
CDK2
CDK4
CDK6
EP300
KIF15
−1
1

Figure S3

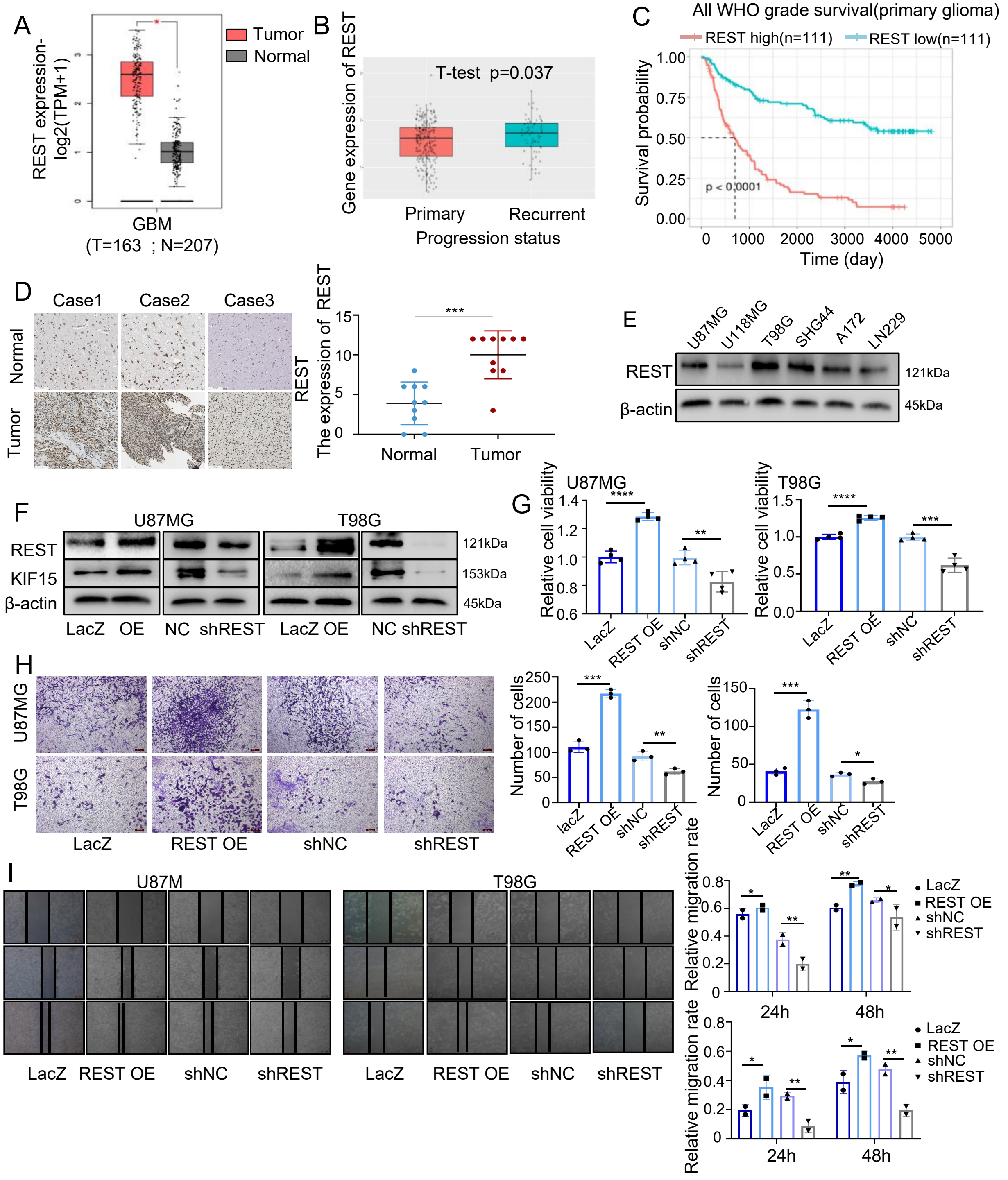
A
REST expression-log2(TPM+1)
Tumor
Normal
GBM
(T=163 ; N=207)
B
T-test p=0.037
Gene expression of REST
Primary
Recurrent
Progression status
C
All WHO grade survival(primary glioma)
REST high(n=111)
REST low(n=111)
Survival probability
p < 0.0001
Time (day)
D
Case1
Case2
Case3
Normal
Tumor
The expression of REST
Normal
Tumor
E
U87MG
U118MG
T98G
SHG44
A172
LN229
REST
β-actin
121kDa
45kDa
F
U87MG
T98G
REST
KIF15
β-actin
121kDa
153kDa
45kDa
LacZ OE NC shREST LacZ OE NC shREST
G
U87MG
T98G
Relative cell viability
LacZ
REST OE
shNC
shREST
H
U87MG
T98G
LacZ
REST OE
shNC
shREST
Number of cells
I
U87M
T98G
0h
24h
48h
LacZ REST OE shNC shREST
Relative migration rate
24h
48h
LacZ
REST OE
shNC
shREST

Figure S4

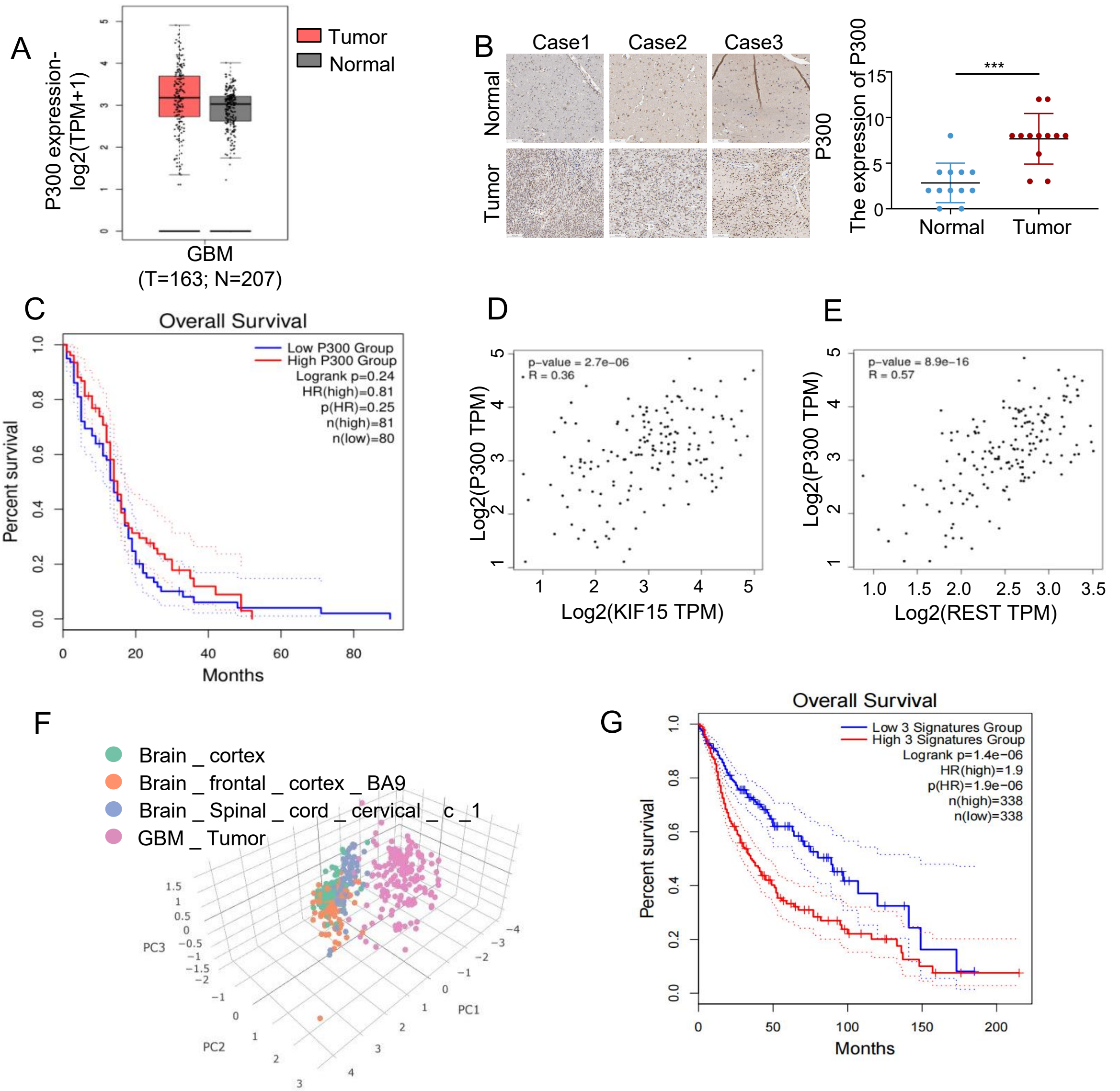

A
P300 expression-log2(TPM+1)
Tumor
Normal
GBM
(T=163; N=207)
B
Case1
Case2
Case3
Normal
Tumor
P300
The expression of P300
***
Normal
Tumor
C
Overall Survival
Low P300 Group
High P300 Group
Logrank p=0.24
HR(high)=0.81
p(HR)=0.25
n(high)=81
n(low)=80
Percent survival
Months
D
p-value = 2.7e-06
R = 0.36
Log2(P300 TPM)
Log2(KIF15 TPM)
E
p-value = 8.9e-16
R = 0.57
Log2(P300 TPM)
Log2(REST TPM)
F
Brain _ cortex
Brain _ frontal _ cortex _ BA9
Brain _ Spinal _ cord _ cervical _ c _ 1
GBM _ Tumor
PC1
PC2
PC3
G
Overall Survival
Low 3 Signatures Group
High 3 Signatures Group
Logrank p=1.4e-06
HR(high)=1.9
p(HR)=1.9e-06
n(high)=338
n(low)=338
Percent survival
Months
